# Supramolecular multi-electron redox photosensitisers comprising a ring-shaped Re(i) tetranuclear complex and a polyoxometalate†

**DOI:** 10.1039/d2sc04252e

**Published:** 2022-12-01

**Authors:** Maria Takahashi, Tsuyoshi Asatani, Tatsuki Morimoto, Yoshinobu Kamakura, Kotaro Fujii, Masatomo Yashima, Naoki Hosokawa, Yusuke Tamaki, Osamu Ishitani

**Affiliations:** a Department of Chemistry, School of Science, Tokyo Institute of Technology O-okayama 2-12-1-NE-1 Meguro-ku Tokyo 152-8550 Japan ishitani@chem.titech.ac.jp; b School of Engineering, Tokyo University of Technology 1404-1 Katakura Hachioji Tokyo 192-0982 Japan; c Department of Chemistry, Graduate School of Advanced Science and Engineering, Hiroshima University 1-3-1 Kagamiyama Higashi-Hiroshima Hiroshima 739 8526 Japan

## Abstract

Redox photosensitisers (PSs) play essential roles in various photocatalytic reactions. Herein, we synthesised new redox PSs of 1 : 1 supramolecules that comprise a ring-shaped Re(i) tetranuclear complex with 4+ charges and a Keggin-type heteropolyoxometalate with 4− charges. These PSs photochemically accumulate multi-electrons in one molecule (three or four electrons) in the presence of an electron donor and can supply electrons with different reduction potentials. PSs were successfully applied in the photocatalytic reduction of CO_2_ using catalysts (Ru(ii) and Re(i) complexes) and triethanolamine as a reductant. In photocatalytic reactions, these supramolecular PSs supply a different number of electrons to the catalyst depending on the redox potential of the intermediate, which is made from the one-electron-reduced species of the catalyst and CO_2_. Based on these data, information on the reduction potentials of the intermediates was obtained.

## Abstract 2

Supramolecular photosensitisers comprising a ring-shaped Re(i) tetranuclear complex and a Keggin-type heteropolyoxometalate were synthesised. They can photochemically accumulate four electrons and supply them for the photocatalytic CO_2_ reduction.

## Graphical abstract

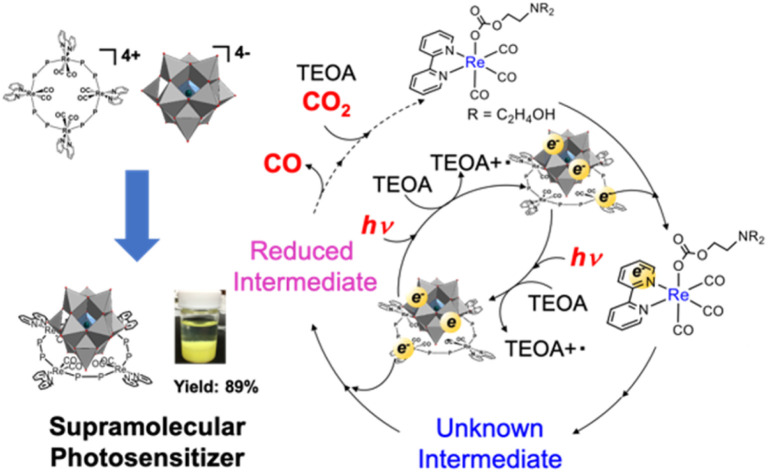

## Introduction

Redox reactions initiated by photochemical electron transfer have been widely used in various research fields, such as organic synthesis^1,2^ and artificial photosynthesis.^3–7^ In these reactions, redox photosensitisers (PSs), also referred to as photoredox catalysts,^1,2^ induce photochemical electron transfer from an electron donor to an acceptor as a starting process.^7^ For example, the triplet metal-to-ligand-charge-transfer (^3^MLCT) excited state of tris(2,2′-bipyridine) ruthenium(ii) ([Ru(bpy)_3_]^2+^) is formed through photoirradiation; this excited state has superior reduction and oxidation capabilities, *i.e.*, 2.12 eV greater (as excitation energy) compared to its ground state.^8^ Since both the oxidised and reduced states of the Ru complex are stable, they are used as effective PSs in numerous photochemical redox reactions. In recent years, Ir(iii) complexes,^1,7,9,10^ Os(ii) complexes,^7,11^ and Cu(i) heteroleptic complexes^12–18^ and some organic compounds^2*b*,19–21^ have also been used as PSs.

Generally, the photoexcitation of a molecule can induce only one-electron transfer. Hence, typical PSs can initiate only a one-electron transfer from the electron donor to the acceptor. Since, on the other hand, one-electron reduction of CO_2_ requires very high amounts of energy (*E*^0^ = −1.9 V *vs.* NHE), the two-electron reduction of CO_2_ coupled with another chemical reaction, such as proton addition can be applied to lower the required energy to produce stable products such as CO (*E*^0^ = −0.53 V) and HCOOH (*E*^0^ = −0.61 V). Hence, catalysts that completely accept two electrons through redox-photosensitised reaction(s) and reduce CO_2_ should be used with PSs to achieve efficient photocatalytic CO_2_ reduction.^7^

In such two-component photocatalytic systems, an intermediate produced by the chemical reaction(s) of the one-electron-reduced species (OERS) of the catalyst must rapidly accept one more electron because the side reactions of the active intermediate induce decomposition of the catalyst and lower the durability of the photocatalytic system. For avoiding this decomposition process of the catalyst, a significantly higher number of PSs than catalysts has been used in many reported photocatalytic systems to suppress these side reactions because the usual PSs initiate only a one-electron transfer.^7^ In these systems, however, the decomposition of PSs cannot be avoided owing to the photochemical decomposition of the OERSs of the excess PSs, which accumulate in the reaction solution.

Therefore, developing PSs that can accumulate multi-electrons in one molecule and donate them to the catalytic reaction with suitable timing should inspire a new direction in photocatalytic redox reactions. However, only a limited number of PSs can accumulate multi-electrons. For example, Ru(ii) complexes with quinone or pyridinium-cation moieties integrated into ligands^22^ or viologen moieties attached to ligands^23^ have been reported. However, such PSs have weak reduction power, and their application in photocatalytic reactions has been limited to low-energy reactions, such as H_2_ evolution.^23–27^ Another challenge associated with multi-electron-accumulating PSs is that the accumulated electrons in PSs with multiple numbers of the same or similar photosensitiser units in one molecule, such as ring-shaped Re(i) multinuclear complexes,^28,29,30*a*,37^ have the same or similar reduction powers. It cannot accumulate multi-electrons in the presence of a catalyst because its OERS rapidly passes an accepted electron to the catalyst, *i.e.*, it works only as a one-electron transfer photosensitiser.^28,29^

In photocatalytic systems for CO_2_ reduction, the reduction potentials of the catalyst and the intermediate derived from the reaction of the reduced catalyst with a substrate such as CO_2_ should be different. Since the reduction potential of the intermediate, which is very important information for developing efficient photocatalytic systems, is often more positive than the first one owing to the following chemical reaction or reactions, the reduction potential cannot be determined using ordinary electrochemical methods. To the best of our knowledge, there have been no reports of a multi-electron-accumulating PS that can precisely supply two electrons to the catalyst and intermediate in photocatalytic CO_2_ reduction reactions.

**Chart 1:**
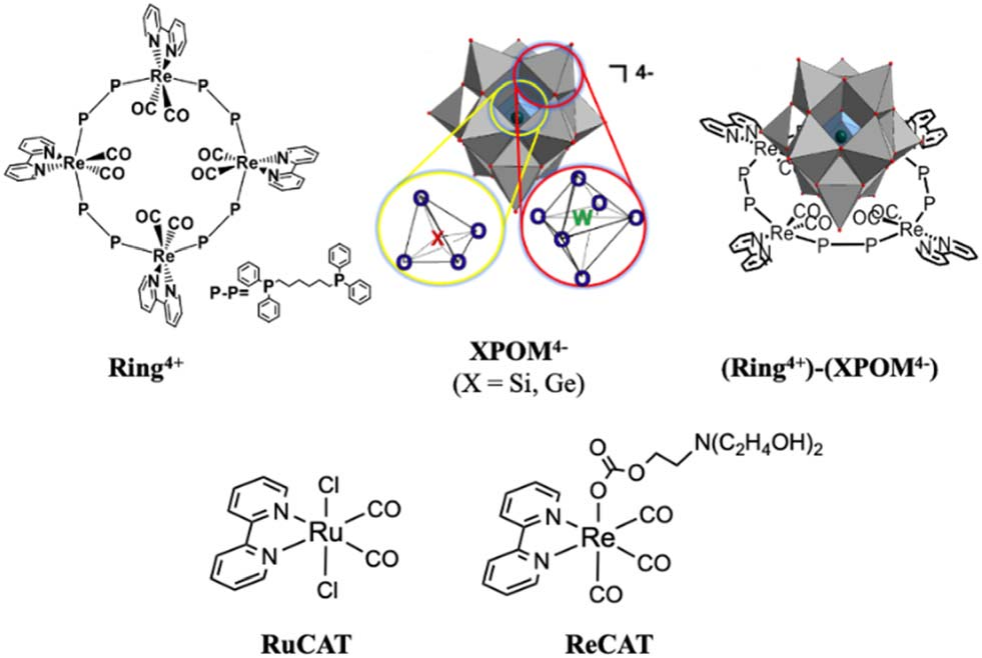
Structures of Ring^4+^, XPOM^4−^, (Ring^4+^)–(XPOM^4−^) (X = Si, Ge), RuCAT, and ReCAT.

Herein, we report the first examples of supramolecular redox photosensitisers consisting of a ring-shaped Re(i) tetranuclear complex (Ring^4+^) and Keggin-type heteropolyoxometalate [XW_12_O_40_]^4−^ (XPOM^4−^, X = Si, Ge) (Chart 1). Ring^4+^ has four positive charges owing to the one plus charge of each Re(i) unit and the space inside the ring structure.^28–30*a*^ Its lowest ^3^MLCT excited state has a long lifetime even in solution at 25 °C (*τ* = 225 and 406 ns), of which the dual phosphorescence property is attributed to stable conformers,^28^ and high oxidation power. Its OERS is relatively stable, and its reduction power is high. In contrast, XPOM^4−^, in which twelve octahedral tungsten oxyanions surround a central silicate or germanate group, has four negative charges. They can electrochemically accumulate multi-electrons in a single molecule because their reduced states are stable.^31–33^

We found that strong electrostatic interactions between XPOM^4−^ and Ring^4+^ formed a 1 : 1 ion pair (Ring^4+^)–(XPOM^4−^), as shown in Chart 1. In these supramolecules, the Ring^4+^ unit functions as an intramolecular PS that photochemically transfers multi-electrons to the XPOM^4−^ unit in the presence of triethanolamine in the initial stage. It accumulates one more electron in one of the Re(i) complex moieties, resulting in three electrons with different reduction powers. Using this novel photochemical multi-electron accumulating system as a PS, we developed new photocatalytic CO_2_ reduction systems accompanied by Re(i)- or Ru(ii)-complex catalysts (RuCAT and ReCAT in Chart 1). In addition, a comparison of the photosensitising abilities of the two types of (Ring^4+^)–(XPOM^4−^), where X = Si or Ge, provides information on redox potentials of the intermediates formed from the OERSs of the Re and Ru catalysts.

## Results and discussion

### Synthesis of (Ring^4+^)–(XPOM^4−^) and their structures

(Ring^4+^)(PF_6_^−^)_4_^30^ and (TBA^+^)_4_(XPOM^4−^) (X = Si or Ge; TBA^+^ = tetrabutylammonium cation)^34,35^ were synthesised according to previously reported procedures. An acetonitrile solution (6 mL) containing (Ring^4+^)(PF_6_^−^)_4_ (3.00 μmol) was added to another acetonitrile solution containing (TBA^+^)_4_(SiPOM^4−^) (3.00 μmol), giving (Ring^4+^)–(SiPOM^4−^) as yellow solids in a 96% yield (Fig. S1†). A similar method, except for the usage of (TBA^+^)_4_(GePOM^4−^) instead of (TBA^+^)_4_(SiPOM^4−^), gave (Ring^4+^)–(GePOM^4−^) also as yellow solids (yield = 97%). Their elemental analysis data clearly indicate that both of them were 1 : 1 ion pairs consisting of Ring^4+^ and XPOM^4−^.

**Fig. 1:**
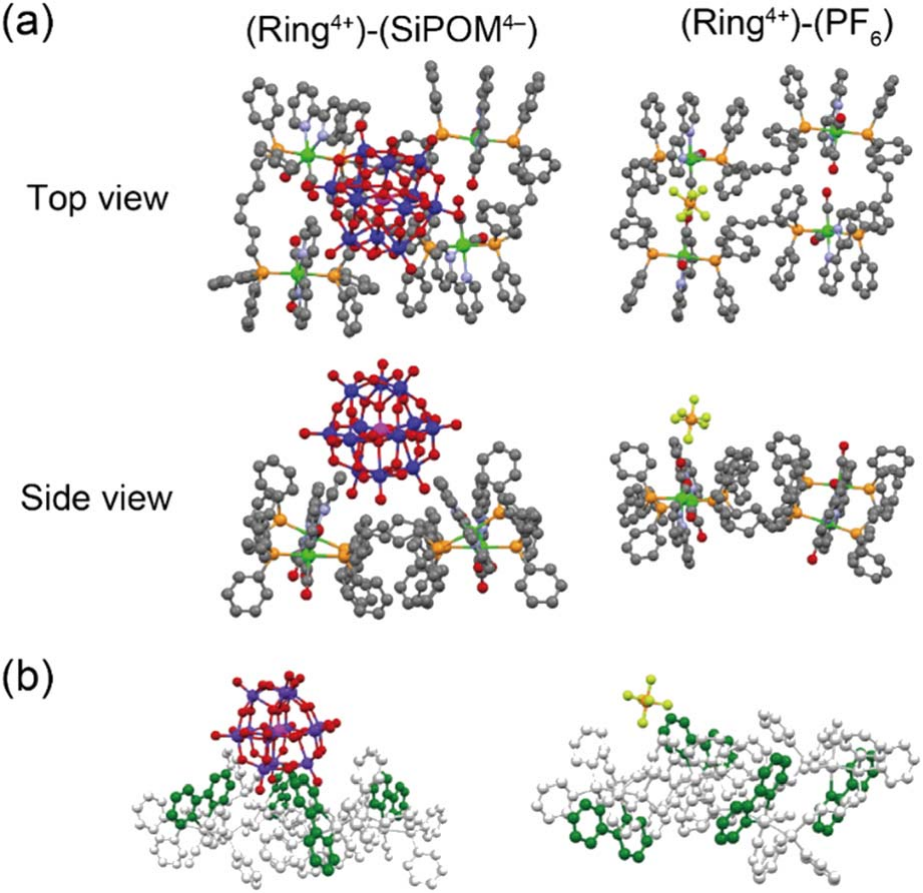
(a) Crystal structures of (Ring^4+^)–(SiPOM^4−^) and (Ring^4+^)(PF_6_^−^)_4_ in top and side view. The grey, light-blue, red, orange, yellow, magenta, violet, and green spheres represent C, N, O, P, F, Si, W, and Re atoms, respectively. H atoms omitted for clarity. (b) Spatial orientation of the bpy ligands (highlighted in green).

Single crystals of both (Ring^4+^)–(SiPOM^4−^), and (Ring^4+^)(PF_6_^−^)_4_ were obtained by recrystallisation using *N*,*N*-dimethylacetamide/methanol (crystallographic data of (Ring^4+^)–(SiPOM^4−^) and (Ring^4+^)(PF_6_^−^)_4_ are shown in Table S1†). Fig. 1a shows the crystal structures determined using single-crystal X-ray diffraction, which shows that Ring^4+^ and SiPOM^4−^ form 1 : 1 ion pairs. There were observable differences between the Ring^4+^ moieties of (Ring^4+^)–(SiPOM^4−^) and (Ring^4+^)(PF_6_^−^)_4_.

For instance, the distances between two adjacent Re(i) ions in (Ring^4+^)–(SiPOM^4−^) (7.77, 11.41, 7.77, 11.41 Å) were smaller than those in (Ring^4+^)(PF_6_^−^)_4_ (7.99, 11.28, 8.12, 11.67 Å). All the bpy ligands in (Ring^4+^)–(SiPOM^4−^) were seated on the same side of the least-squares plane of the four Re(i) units in the same Ring^4+^. The SiPOM^4−^ moiety was seated and closely surrounded by the four bpy units (Fig. 1b, left). In contrast, in (Ring^4+^)(PF_6_^−^)_4_, the two bpy units on the diagonal were seated on the same side, while the other two were on the opposite side (Fig. 1b, right). These results strongly indicate that all Re(i) ions with a positive charge and all bpy ligands that have a positive charge compared to the CO ligands owing to their different electron-withdrawing properties should compensate for the four negative charges of the SiPOM^4−^ moiety. The closest distance between the Ring^4+^ and SiPOM^4−^ in (Ring^4+^)–(SiPOM^4−^) was 3.26 Å for the C(bpy) and O(SiPOM^4−^), suggesting that interaction between Ring^4+^ and SiPOM^4−^ exist because this distance is similar to the sum of the van der Waals radii of carbon (1.70 Å) and oxygen (1.52 Å).^36^

### (Ring^4+^)–(XPOM^4−^) dissolved in DMSO solution

Both (Ring^4+^)–(SiPOM^4−^) and (Ring^4+^)–(GePOM^4−^) were soluble in dimethyl sulfoxide (DMSO); however, only in small amounts in acetonitrile and *N*,*N*-dimethylformamide. ^1^H NMR spectra of (Ring^4+^)–(SiPOM^4−^) and (Ring^4+^)(PF_6_^−^)_4_ in DMSO-*d*_6_ were different (Fig. S2, Table S2†). The peaks attributed to the protons at the 4,4′-positions (bpy-4 in Table S2†) of (Ring^4+^)–(SiPOM^4−^) broadened, and about 0.4 ppm downfield shifted compared to those of (Ring^4+^)(PF_6_^−^)_4_. The other peaks of the bpy ligand and phenyl groups of the phosphine ligands of (Ring^4+^)–(SiPOM^4−^) were slightly shifted (−0.09 to 0.09 ppm). The FT-IR spectra of (Ring^4+^)–(SiPOM^4−^) and (Ring^4+^)(PF_6_^−^)_4_ measured in DMSO were also different (Fig. S3a†): *ν*_CO_ = 1927 and 1846 cm^−1^ in the solution of (Ring^4+^)(PF_6_^−^)_4_ while *ν*_CO_ peaks at 1928 and 1852 cm^−1^ in the solution of (Ring^4+^)–(SiPOM^4−^), whose shapes were also different. These differences in the spectra indicate that Ring^4+^ and SiPOM^4−^ dissolved in the DMSO solution interact closely, even in solution. ^1^H NMR and FT-IR spectra of (Ring^4+^)–(GePOM^4−^) in DMSO-*d*_6_ and DMSO, respectively, also showed similar differences from those of (Ring^4+^)(PF_6_^−^)_4_ (Table S2, Fig. S3b†).

**Fig. 2:**
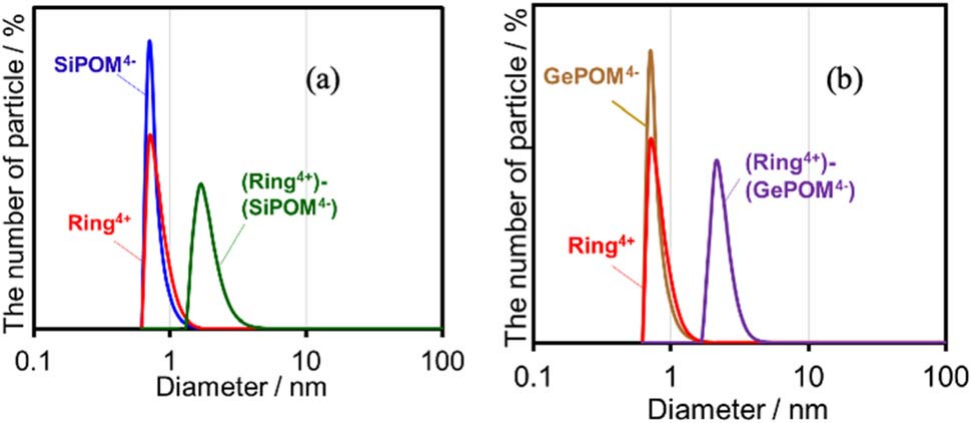
Particle size distributions of the DMSO solutions containing (a) (Ring^4+^)(PF_6_^−^)_4_, (TBA^+^)_4_(SiPOM^4−^), or (Ring^4+^)–(SiPOM^4−^), and (b) (Ring^4+^)(PF_6_^−^)_4_, (TBA^+^)_4_(GePOM^4−^), or (Ring^4+^)–(GePOM^4−^).

Since Ring^4+^ and XPOM^4−^ interact with each other even in DMSO solutions, we measured particle sizes in DMSO solutions containing (Ring^4+^)(PF_6_^−^)_4_, (TBA^+^)_4_(SiPOM^4−^), (Ring^4+^)–(SiPOM^4−^), (TBA^+^)_4_(GePOM^4−^), or (Ring^4+^)–(GePOM^4−^) using dynamic light scattering (DLS). Fig. 2 shows the particle size distributions. In the solutions of (Ring^4+^)(PF_6_^−^)_4_, (TBA^+^)_4_(SiPOM^4−^), and (TBA^+^)_4_(GePOM^4−^), small particles with diameters (*D*) of approximately 1 nm were mainly observed, and these are attributable to solvated monomeric Ring^4+^, SiPOM^4−^, and GePOM^4−^, respectively, some of which may interact with the counter anions or cations because of the size similarity to the X-ray structure of each ion of (Ring^4+^)–(SiPOM^4−^) (Fig. 1). In contrast, in the solution containing (Ring^4+^)–(SiPOM^4−^), larger particles (2.9 ± 0.4 nm) were mainly observed compared to those of Ring^4+^ and SiPOM^4−^ (Fig. 2a). Therefore, even in the DMSO solution, Ring^4+^ and SiPOM^4−^ maintained the interaction and formed a supramolecule consisting of one molecule of Ring^4+^ and one molecule of SiPOM^4−^. In the DMSO solution of (Ring^4+^)–(GePOM^4−^), particles of similar size (3.2 ± 0.5 nm) were observed (Fig. 2b). Therefore, it can be deduced that strong electrostatic interactions maintain supramolecular interactions between Ring^4+^ and XPOM^4−^ even in DMSO solutions and mainly form a 1 : 1 supramolecule consisting of one ion each.

**Fig. 3:**
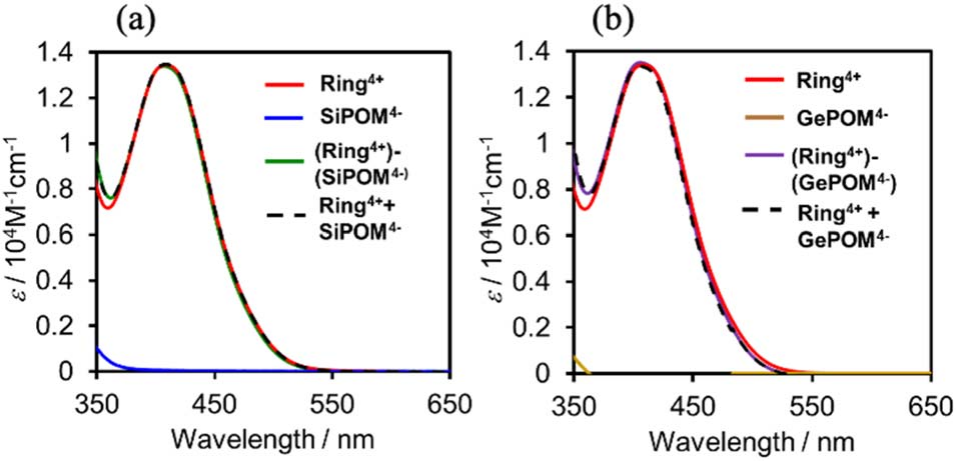
UV-vis absorption spectra of (a) (Ring^4+^)–(SiPOM^4−^) and (b) (Ring^4+^)–(GePOM^4−^) in the DMSO solutions: spectra of (Ring^4+^)(PF_6_^−^)_4_, (TBA^+^)_4_(XPOM^4−^), and summation of them were also shown.

Fig. 3 shows UV-vis absorption spectra of (Ring^4+^)–(XPOM^4−^) and their constituent ions in DMSO solutions. The similarity in the spectra of (Ring^4+^)–(XPOM^4−^) and the summation of those of (Ring^4+^)(PF_6_^−^)_4_ and (TBA^+^)_4_(XPOM^4−^) indicates that there was no strong electric interaction between the Ring^4+^ and XPOM^4−^ units of (Ring^4+^)–(XPOM^4−^) in the ground states, although they exist closely in the solutions as described above. In both spectra of (Ring^4+^)–(XPOM^4−^), a broad peak was observed at *λ*_max_ = 408 nm, which is attributed to singlet metal-to-ligand-charge-transfer (^1^MLCT) absorption of the Ring^4+^ unit.

In the much shorter wavelength region, singlet ligand-to-metal-charge-transfer (^1^LMCT) absorption of the XPOM^4−^ unit and singlet π–π* absorption of the Ring^4+^ unit were observed. Therefore, only the Ring^4+^ unit of the supramolecule can be selectively excited by light at *λ*_ex_ ≥ 400 nm in photochemical reactions.

**Fig. 4:**
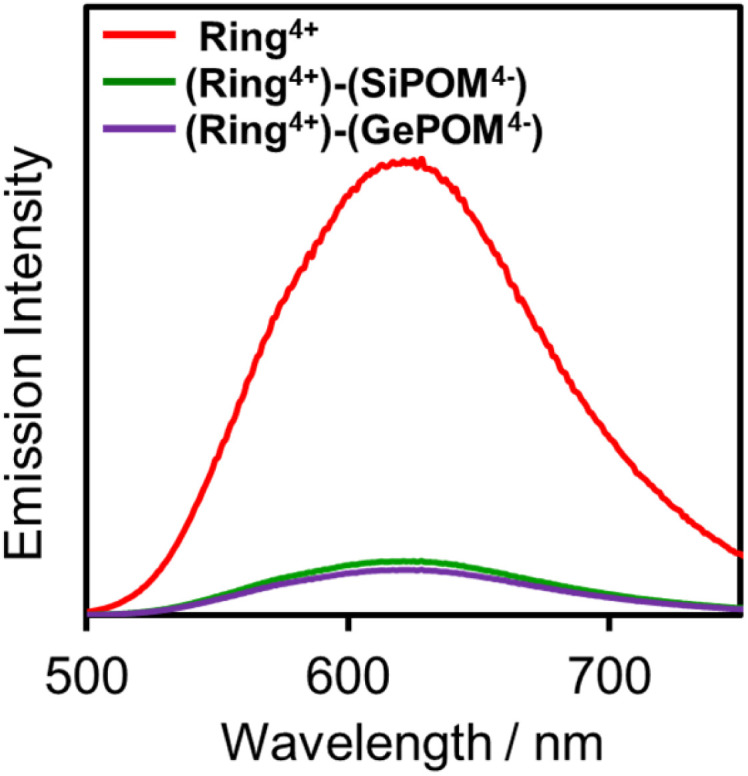
Emission spectra of (Ring^4+^)(PF_6_^−^)_4_ (red), (Ring^4+^)–(SiPOM^4−^) (green), and (Ring^4+^)–(GePOM^4−^) (purple): *λ*_ex_ = 400 nm, concentration of the complex was 0.05 mM.

Fig. 4 shows emission spectra of (Ring^4+^)(PF_6_^−^)_4_ and (Ring^4+^)–(XPOM^4−^) dissolved in DMSO solutions using excitation light at *λ*_ex_ = 400 nm. This outcome is due to the emission from Ring^4+^ or the Ring^4+^ unit of the supramolecules because the shapes of the emission spectra were very similar in all the solutions. However, the strengths of both (Ring^4+^)–(SiPOM^4−^) and (Ring^4+^)–(GePOM^4−^) were significantly weaker than that of (Ring^4+^)(PF_6_^−^)_4_. The photophysical properties of the samples are summarised in Table 1. The emission quantum yields (*Φ*_em_) of the Ring^4+^ units in (Ring^4+^)–(SiPOM^4−^) and (Ring^4+^)–(GePOM^4−^) were only 15% and 13% compared to that of (Ring^4+^)(PF_6_^−^)_4_, respectively. Therefore, it can be inferred that most of the ^3^MLCT excited states of the Ring^4+^ units in the supramolecules were quenched by the XPOM^4−^ unit.

**Table 1:** Photophysical properties^a^

Material	*λ*_ab_max/nm (*ε*/M^−1^ cm^−1^)	*λ*_em_ b/nm	*Φ*_em_ b/%
(Ring^4+^)(PF_6_^−^)_4_	409 (13 300)	623	4.6
(Ring^4+^)–(SiPOM^4−^)	408 (13 000)	624	0.7
(Ring^4+^)–(GePOM^4−^)	408 (13 500)	624	0.6

a Solvent: DMSO.

b *λ*_em_ = 400 nm, concentration of the complex = 0.05 mM.

**Fig. 5:**
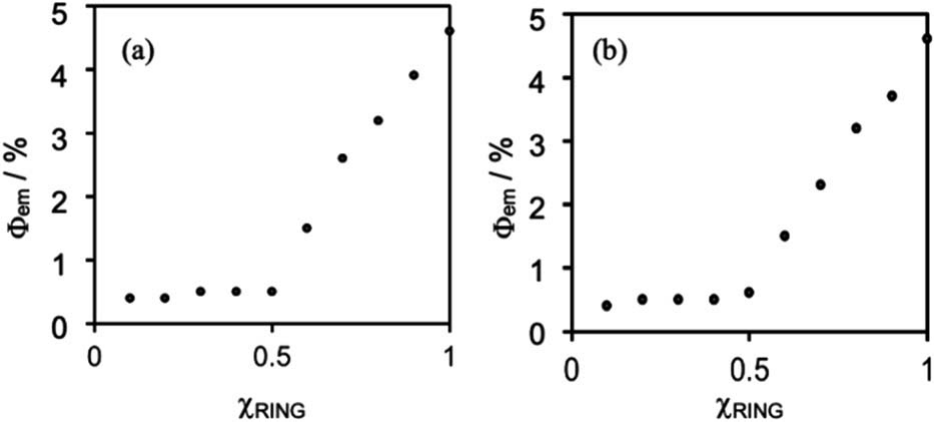
Job plots of emission quantum yields of the Ring^4+^ in DMSO solutions in the absence or presence of (a) (TBA^+^)_4_(SiPOM^4−^) and (b) (TBA^+^)_4_(GePOM^4−^). *χ*_RING_= [Ring^4+^]/([Ring^4+^] + [XPOM^4−^]) where [Ring^4+^] + [XPOM^4−^] = 0.10 mM.

Fig. 5 shows Job plots of emission quantum yields (*Φ*_em_) from the mixed solutions of the DMSO solutions containing (Ring^4+^)(PF_6_^−^)_4_ and (TBA^+^)_4_(XPOM^4−^). Their total amount was the same (0.10 mM) but their ratio (*χ*_RING_ = [Ring^4+^]/([Ring^4+^] + [XPOM^4−^])) was changed. At *χ*_RING_ > 0.5, smaller *χ*_RING_ made higher *Φ*_em_, but *Φ*_em_ was almost constant at *χ*_RING_ ≤ 0.5. These results clearly indicate that almost all of the added XPOM^4−^ were converted into the supramolecule with Ring^4+^ in the DMSO solution when [(TBA^+^)_4_(XPOM^4−^)] < [(Ring^4+^)(PF_6_^−^)_4_]. Even on the occasion of [(TBA^+^)_4_(XPOM^4−^)] = [(Ring^4+^)(PF_6_^−^)_4_], most of Ring^4+^ interacted with XPOM^4−^ to form the 1 : 1 supramolecule (Ring^4+^)–(XPOM^4−^) in both DMSO solutions containing either (TBA^+^)_4_(GePOM^4−^) or (TBA^+^)_4_(SiPOM^4−^).

**Fig. 6:**
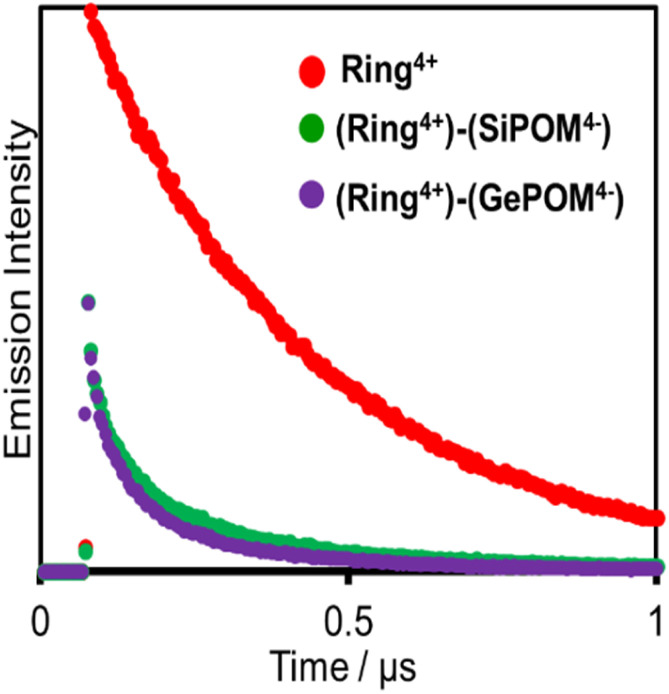
Emission decays of Ring^4+^ (red), (Ring^4+^)–(SiPOM^4−^) (green), and (Ring^4+^)–(GePOM^4−^) (purple): *λ*_ex_ = 400 nm, *λ*_det_ = 615 nm, concentrations of the complexes = 0.05 mM: the number of integration was unified.

Fig. 6 shows emission decays of Ring^4+^ and (Ring^4+^)–(XPOM^4−^) dissolved in the DMSO solutions using the single-photon counting method, normalised by absorbed photon numbers (*λ*_ex_ = 400 nm, *λ*_det_ = 615 nm). Notably, the emission strength at time = 0 was very different between (Ring^4+^)–(SiPOM^4−^) and free Ring^4+^, *i.e.*, the former was much weaker than the latter when the number of integrations was unified. This result clearly indicates that static quenching of the excited state of the Ring^4+^ unit by the SiPOM^4−^ unit rapidly proceeded within the time resolution of the apparatus (200 ps). Based on careful investigation of these emission decay data as described in the ESI section,† we can conclude that the percentage of dissociation of (Ring^4+^)–(SiPOM^4−^) was only 3.9%, and more than 85% of the excited state of the Ring^4+^ unit was statically quenched by the SiPOM^4−^ unit in the DMSO solution dissolving 0.05 mM of (Ring^4+^)–(SiPOM^4−^).

In the DMSO solution containing 0.05 mM of (Ring^4+^)–(GePOM^4−^), a similar emission quenching (Fig. S4†) were observed compared to the free Ring^4+^. These findings show that the supramolecular structure of (Ring^4+^)–(GePOM^4−^) was maintained when the Ring^4+^ unit was excited. Additionally, the ^3^MLCT excited state of the Ring^4+^ unit was efficiently quenched by the GePOM^4−^ unit in the (Ring^4+^)–(GePOM^4−^) as well (more than 87%).

### Electrochemical properties

**Fig. 7:**
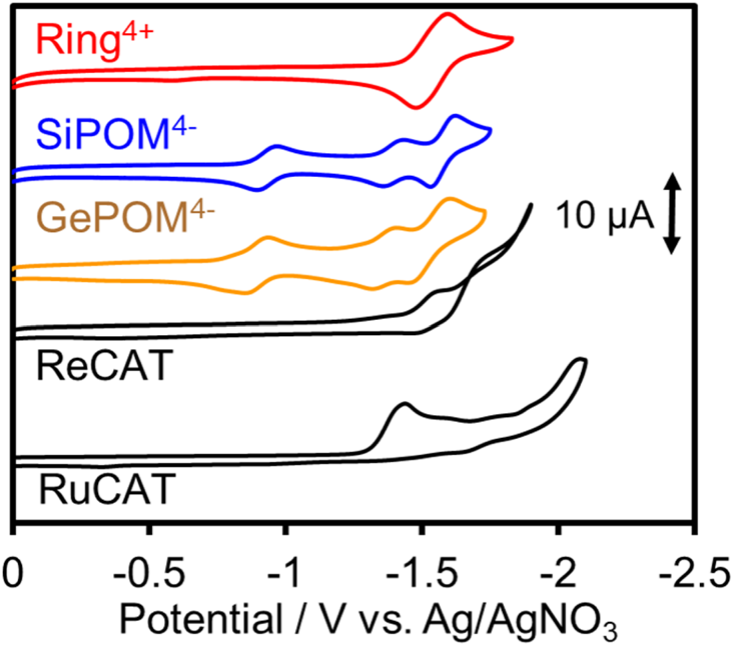
Cyclic voltammograms of DMSO–TEOA (5 : 1 v/v) solutions containing complexes, *i.e.*, (Ring^4+^)(PF_6_^−^)_4_, (TBA^+^)_4_(SiPOM^4−^), (TBA^+^)_4_(GePOM^4−^), Re(bpy)(CO)_3_{OC(O)OC_2_H_4_N(C_2_H_4_OH)} (ReCAT), and Ru(bpy)(CO)_2_Cl_2_ (RuCAT), and (TBA^+^)(PF_6_^−^) as supporting electrolyte measured under CO_2_ atmosphere by using a glassy carbon working electrode, 0.01 mM Ag/AgNO_3_ reference electrode, and a Pt counter electrode with scan rate of 100 mV s^−1^.

Since (Ring^4+^)–(XPOM^4−^)s were partially separated into Ring^4+^ and XPOM^4−^ in solutions containing electrolyte, we separately measured cyclic voltammograms (CVs) of each unit. Since, as described above, the UV-vis absorption spectra does not change between (Ring^4+^)–(XPOM^4−^) and the summation of Ring^4+^ and XPOM^4−^ (1:1), the redox potentials of each unit of (Ring^4+^)–(XPOM^4−^)s should scarcely change compared to those of separated Ring^4+^ and XPOM^4−^. Fig. 7 shows CVs of (Ring^4+^)(PF_6_^−^)_4_ and (TBA^+^)_4_(XPOM^4−^) in DMSO–TEOA (5 : 1 v/v) solutions containing 0.1 M of (TBA^+^)(PF_6_^−^) as supporting electrolyte under a CO_2_ atmosphere. The voltammograms of catalysts used in photocatalytic reactions as described below are also added. In the DMSO solution of (Ring^4+^)(PF_6_^−^)_4_, a reversible redox wave was observed at *E*_1/2_ = −1.54 V *vs.* Ag/AgNO_3_ (Δ*E*_p_ = 118 mV), as previously reported in a DMF solution.^30*a*^ This redox is attributed to the one-electron reduction of each Re unit; that is, four electrons were introduced into Ring^4+^ at once (eqn (1)).

1 [(–C_6_H_12_PPh_2_)Re^I^(bpy)(CO)_2_(PPh_2_–)]_4_^4+^ + 4e^−^ ⇆ [(–C_6_H_12_PPh_2_)Re^I^(bpy^−^˙)(CO)_2_(PPh_2_–)]_4_

In both solutions of (TBA^+^)_4_(XPOM^4−^), three reversible waves were observed at *E*_1/2_ = −0.93 V (Δ*E*_p_ = 77 mV), *E*_1/2_ = −1.40 V (Δ*E*_p_ = 78 mV), and *E*_1/2_ = −1.58 V (Δ*E*_p_ = 90 mV) in the case of SiPOM^4−^, and *E*_1/2_ = −0.88 V (Δ*E*_p_ = 68 mV), *E*_1/2_ = −1.33 V (Δ*E*_p_ = 85 mV), and *E*_1/2_ = −1.50 V (Δ*E*_p_ = 100 mV) in the case of GePOM^4−^, respectively. SiPOM^4−^ can accept one, one, and two electrons in order, that is, four electrons in one molecule, according to the reactions shown in eqn (2)–(4), particularly under acidic conditions.^38^ In the DMSO–TEOA (5 : 1 v/v) solution under a CO_2_ atmosphere, TEOA and contaminated water probably worked as proton donors with the assistance of carbonate produced from the dissolved CO_2_, and carbonate esters produced from CO_2_ and TEOA.^39^ The similarity of the CVs of SiPOM^4−^ and GePOM^4−^ strongly suggests that similar three-step reduction processes (eqn (2)–(4)) also occurred in the CV of GePOM^4−^.

2 XW_12_O_40_^4−^ + e^−^ ⇆ XW_12_O_40_^5−^ (X = Si, Ge)

3 XW_12_O_40_^5−^ + e^−^ ⇆ XW_12_O_40_^6−^

4 XW_12_O_40_^6−^ + 2e^−^ + 2H^+^ ⇆ H_2_XW_12_O_40_^6−^

Although the exact reduction potential of the ^3^MLCT excited state of Ring^4+^ has not been determined because of a lack of information on the excitation energy of Re complexes, it is expected as approximately −1.0 to −1.1 V *vs.* Ag/AgNO_3_.^30^ This is more negative compared to the first reduction potential of SiPOM^4−^ and GePOM^4−^ (*E*_1/2_ = −0.93 V and −0.88 V, respectively). Considering that there is no strong electric interaction between the Ring^4+^ and XPOM^4−^ units, we can conclude that the emission quenching observed in the supramolecule (Ring^4+^)–(XPOM^4−^) proceeds *via* electron transfer from the excited Ring^4+^ unit to XPOM^4−^ (eqn (5)). The reduction potentials described above are listed in Table 2.5
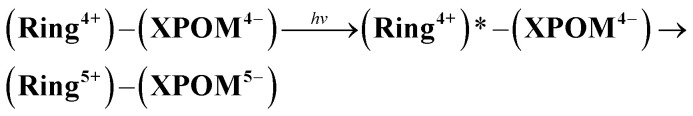


**Table 2:** Redox potentials of the complexes in DMSO–TEOA (5 : 1 v/v) solutions under a CO_2_ atmosphere

Complex	*E* ^red^_1/2_/*V vs.* Ag/AgNO_3_ (Δ*E*/mV)			
	XPOM^4−^/XPOM^5−^	XPOM^5−^/XPOM^6−^	XPOM^6−^/H_2_XPOM^6−^	Ring^4+^/Ring^0^
(Ring^4+^)(PF_6_^−^)_4_	—	—	—	−1.54 (118)
(TBA^+^)_4_(SiPOM^4−^)	−0.93 (77)	−1.40 (78)	−1.58 (90)	—
(TBA^+^)_4_(GePOM^4−^)	−0.88 (68)	−1.33 (85)	−1.50 (100)	—
	*E* ^red^_p_/*V vs.* Ag/AgNO_3_			
RuCAT	−1.42			
ReCAT	−1.50			

**Fig. 8:**
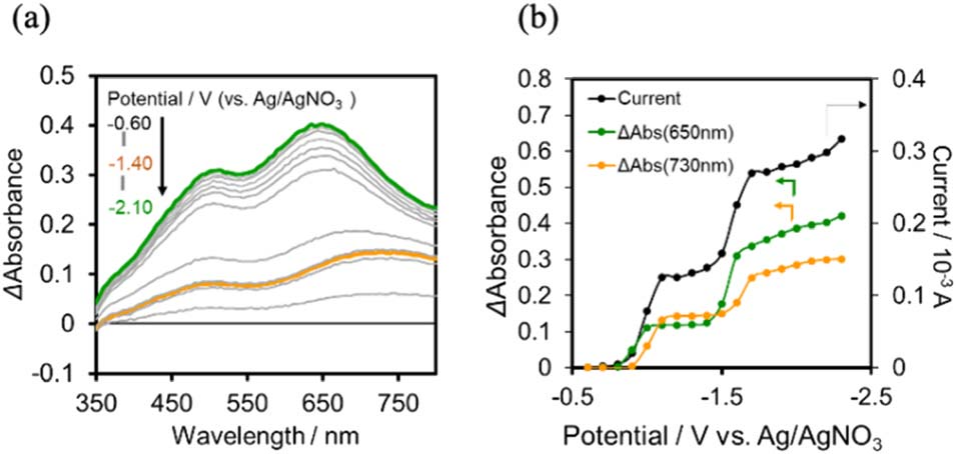
(a) UV-vis absorption changes of the DMSO solution of (TBA^+^)_4_(SiPOM^4−^) (0.5 mM) during the flow electrolysis under an Ar atmosphere, and (b) changes of current (black), absorption change at each potential from that at *E* = −0.6 V (green at *λ* = 650 nm; orange at *λ* = 730 nm): carbon felt working electrode, 0.01 M Ag/AgNO_3_ reference electrode, Pt counter electrode, 0.1 M (TBA^+^)(PF_6_^−^) as an electrolyte, 0.15 mL min^−1^ of the flow rate.

The UV-vis absorption spectra of the reduced XPOM^4−^ were obtained using the flow electrolysis of (TBA^+^)_4_(XPOM^4−^). Fig. 8 shows the results for SiPOM^4−^ measured in DMSO. It indicates a two-step reduction; the first one started at −0.8 V and finalised at −1.1 V and the second one started at −1.4 V and finalised at −1.7 V. They should be attributed to a first one-electron reduction (SiPOM^4−^/SiPOM^5−^) and the second one-electron reduction (SiPOM^5−^/SiPOM^6−^), of which redox potentials are *E*_1/2_ = −0.93 V and −1.40 V as described above (Table 2). The number of electrons injected into one molecule (*n*) at each step is calculated using eqn (6) with a flow rate (*v* = 0.15 mL min^−1^) and the currents to give *n* = 1.0 at *E* = −1.0 V and *n* = 1.1 at *E* = −2.3 V, respectively:6
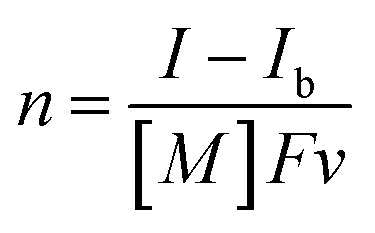
where *I* is current at *E* = −1.1 V or at *E* = −1.7 V, and *I*_b_ is current at *E* = −0.8 V or at *E* = −1.3 V, [*M*] is concentration of (TBA^+^)_4_(SiPOM^4−^), *i.e.*, 0.5 mM, and *F* is the Faraday constant. Fig. 8a shows the difference in the UV-vis absorption spectra of the electrolysis solution at various potentials and at *E* = −0.6 V where the reduction of SiPOM^4−^ did not proceed. New broad absorptions with absorption maxima at *λ* = 480 nm and 730 nm were observed during the first reduction process. These maxima shifted to *λ* = 500 nm and 650 nm after the second reduction. Since these absorption changes were well synchronised with the change in the current (Fig. 8b), we quantitatively determined the absorption spectra of the one- and two-electron reduced species of SiPOM^4−^ (SiPOM^5−^ and SiPOM^6−^). The molar absorption coefficients were *ε*_650_ (SiPOM^5−^) = 1600 M^−1^ cm^−1^ at *λ* = 650 nm, *ε*_730_ (SiPOM^5−^) = 1900 M^−1^ cm^−1^ at *λ* = 730 nm, *ε*_650_ (SiPOM^6−^) = 5400 M^−1^ cm^−1^ at *λ* = 650 nm, and *ε*_730_ (SiPOM^6^^−^) = 4000 M^−1^ cm^−1^ at *λ* = 730 nm.

**Fig. 9:**
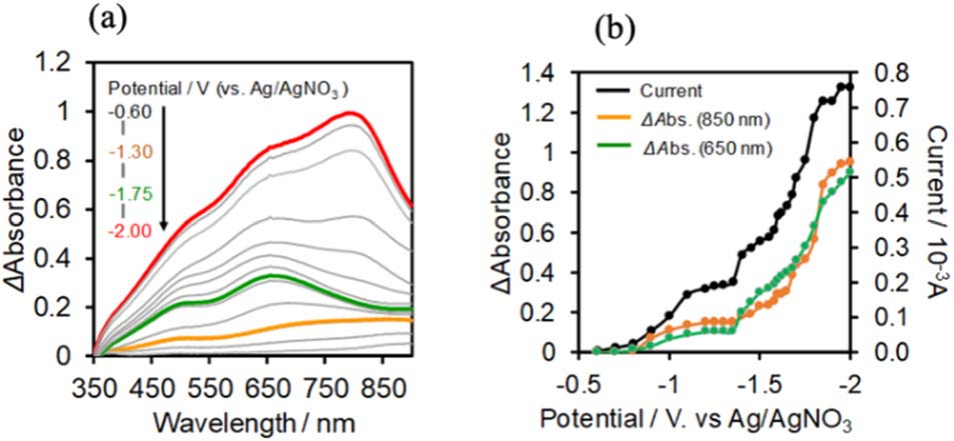
(a) UV-vis absorption changes of the DMSO–EtOH (5 : 1 v/v) solution of (TBA^+^)_4_ (GePOM^4−^) (0.5 mM) during the flow electrolysis under a CO_2_ atmosphere, and (b) changes of current (black), absorption change at each potential from that at *E* = −0.6 V (green at *λ*_ab_ = 650 nm; orange at *λ*_ab_ = 850 nm): carbon felt working electrode, 0.01 M Ag/AgNO_3_ reference electrode, Pt counter electrode, 0.1 M (TBA^+^)(PF_6_^−^) as an electrolyte, 0.15 mL min^−1^ of the flow rate.

In the case of (TBA^+^)_4_(GePOM^4−^), 4-electrons reduction proceeds at a more positive potential than the reduction of Ring^4+^, and this reduction process couples with proton addition (PCET processes). Since the addition of TEOA to DMSO made the viscosity of the solution too high for use in flow electrolysis, ethanol (EtOH) was used as the proton source. A similar CV was obtained in the DMSO–EtOH (5 : 1 v/v) mixed solution containing (TBA^+^)_4_(GePOM^4−^) to that in the DMSO–TEOA (5 : 1 v/v) mixed solution (Fig. S5†). Fig. 9b shows the *I*–*V* curve for the flow electrolysis of (TBA^+^)_4_(GePOM^4−^) in a DMSO–EtOH (5 : 1 v/v) solution, where the current mainly changed in three steps: the current drastically increased at *E* = −0.8 V, −1.45 V, and −1.8 V. In each step, GePOM^4−^ accepts 1.1, 1.0, and 1.8 electron(s), respectively. These results indicate that the three steps of total four-electron reduction proceeded as shown in eqn (2)–(4). Fig. 9a shows the UV-vis absorption spectral changes in the solution during electrolysis.

From these results and investigations, we determined the molar extinction coefficients of the reduced GePOM^4−^ species, that is, *ε*_850_ (GePOM^5−^) = 1900 M^−1^ cm^−1^ (*λ* = 850 nm), *ε*_650_ (GePOM^5−^) = 1400 M^−1^ cm^−1^ (*λ* = 650 nm), *ε*_850_ (GePOM^6−^) = 2200 M^−1^ cm^−1^ (*λ* = 850 nm), *ε*_650_ (GePOM^6−^) = 4000 M^−1^ cm^−1^ (*λ* = 650 nm), *ε*_850_ (H_2_GePOM^6−^) = 9300 M^−1^ cm^−1^ (*λ* = 850 nm), and *ε*_650_ (H_2_GePOM^6−^) = 11 000 M^−1^ cm^−1^ (*λ* = 650 nm).

**Fig. 10:**
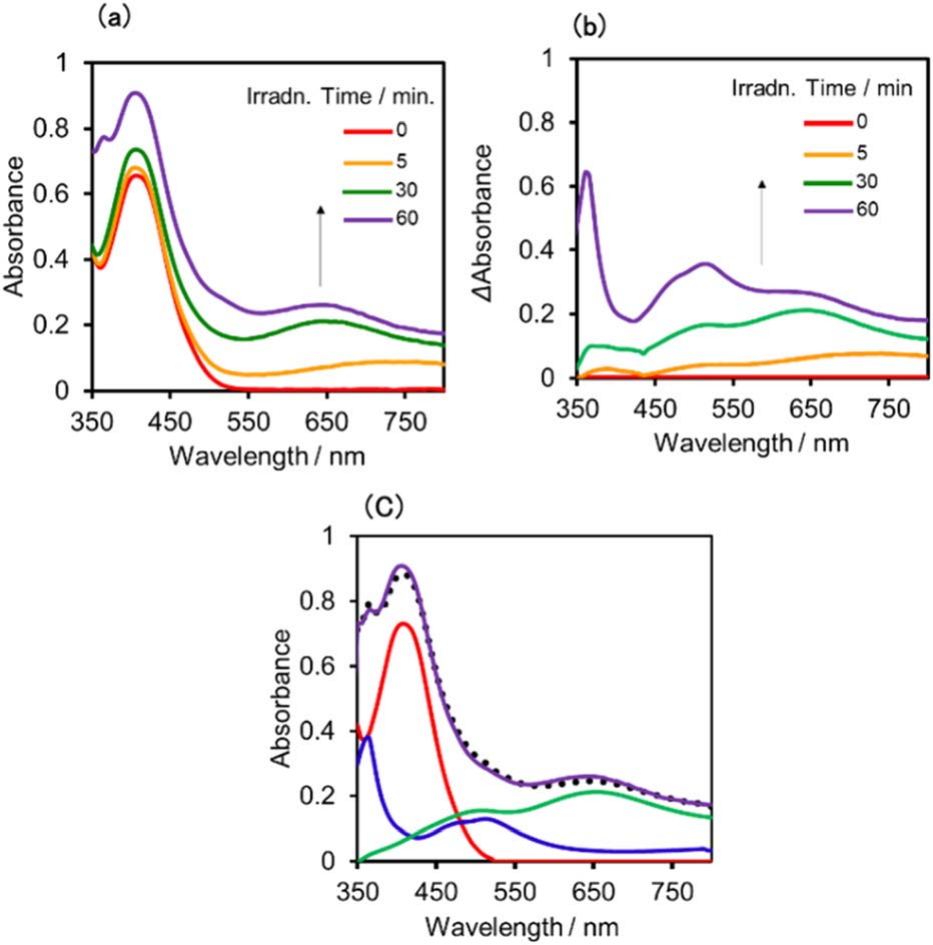
(a) UV-vis absorption spectra changes of the DMSO–TEOA (5 : 1 v/v) solution containing (Ring^4+^)–(SiPOM^4−^) (0.05 mM) during irradiation at *λ*_ex_ = 436 nm (light intensity: 5 × 10^−9^ einstein s^−1^) under a CO_2_ atmosphere, and (b) their deference spectra between before and after irradiation: irradiation time: 5 min (orange line), 30 min (green line), 60 min (purple line). (c) UV-vis absorption spectrum after irradiation for 60 min and its fitting result using the spectra of Ring^4+^ (red line), SiPOM^6−^ (green line), and Ring^3+^ (blue line).

We can use the absorption spectrum of the OERS of the model mononuclear complex *cis*-(CO)-[Re(bpy)(CO)_2_(PEtPh_2_)_2_]^+^ (blue line in Fig. 10c: *λ*_max_ = 362 nm, 490 nm, 515 nm) as that of the OERS of Ring^4+^ because the spectrum of the photochemically reduced Ring^4+^ was well fitted by using the spectra of *cis*-(CO)-[Re(bpy)(CO)_2_(PEtPh_2_)_2_]^+^ and its OERS.^30*a*^

### Photochemical reduction of (Ring^4+^)–(XPOM^4−^)

A DMSO–TEOA (5 : 1 v/v) solution containing (Ring^4+^)–(SiPOM^4−^) was irradiated at *λ*_ex_ = 436 nm with a light intensity of 5 × 10^−9^ einstein s^−1^. Fig. 10a and b show the spectral changes during irradiation (irradiation times of 5 min, 30 min, and 60 min) and differential spectra before and after the irradiation, respectively. Immediately after irradiation, a new broadband with absorption maxima was observed at *λ*_max_ = 480 nm and 730 nm. This result was attributed to the formation of OERS of SiPOM^4−^ as it had a spectrum similar to that of SiPOM^5−^ obtained by flow electrolysis (orange line in Fig. 8a). Further irradiation causes a change in the absorption shape. For example, after irradiation for 30 min, the absorption maxima became *λ*_max_ at 500 nm and 650 nm, which are attributed to the two-electron reduced species (TWERS) of the SiPOM^4−^ unit because of the similarity of the spectrum to that of SiPOM^6−^ (green line in Fig. 8a). Irradiation periods longer than 30 min caused another spectrum change with new absorption maxima at *λ*_max_ = 360 nm, 490 nm (sh), and 515 nm (60 min irradiation: purple line in Fig. 10b).

This outcome was due to the additional formation of OERS of the Ring^4+^ unit (blue line in Fig. 10c),^30*a*^ that is, the three-electron reduced species of the supramolecule ([(Ring^3+^)–(SiPOM^6−^)]^3−^) was produced. All the observed spectra during irradiation can be well fitted by the spectra of Ring^4+^, SiPOM^4−^, SiPOM^5−^, SiPOM^6−^, and/or Ring^3+^. Fig. 10c shows a typical example of the fitting result of the spectrum after 60 min of irradiation, in which SiPOM^6−^, Ring^4+^, and Ring^3+^ were used.

The number of accumulated electrons in one molecule of (Ring^4+^)–(SiPOM^4−^) was obtained as ∼2.6 after irradiation for 60 min through the fitting result. This result indicates that the irradiation of (Ring^4+^)–(SiPOM^4−^) in the presence of TEOA as a reductant caused the stepwise injection of two electrons into the SiPOM^4−^ unit (eqn (7) and (8)), and then one more electron was introduced into the Ring^4+^ unit, giving a three-electron reduced species, that is, [(Ring^3+^)–(SiPOM^6−^)]^3−^ (eqn (9)).

7 [(Ring^4+^)–(SiPOM^4−^)] + e^−^ → [(Ring^4+^)–(SiPOM^5−^)]^−^

8 [(Ring^4+^)–(SiPOM^5−^)]^−^ + e^−^ → [(Ring^4+^)–(SiPOM^6−^)]^2−^

9 [(Ring^4+^)–(SiPOM^6−^)]^2−^ + e^−^ → [(Ring^3+^)–(SiPOM^6−^)]^3−^

**Fig. 11:**
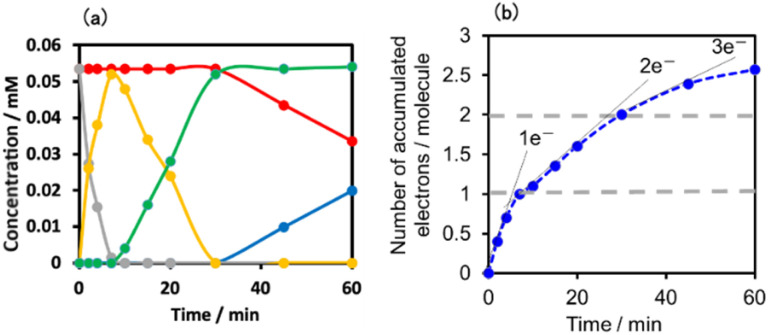
(a) Concentrations of the Ring^4+^ (red line) and SiPOM^4−^ (gray line) units and the reduced species of each unit (SiPOM^5−^ (yellow line), SiPOM^6−^ (green line), and Ring^3+^ (blue line)) during irradiation to a DMSO–TEOA (5 : 1 v/v) solution containing (Ring^4+^)–(SiPOM^4−^) at *λ*_ex_ = 436 nm. (b) Accumulated electrons in one molecule of (Ring^4+^)–(SiPOM^4−^).

Fig. 11a shows the concentrations of the various redox states of each unit in this supramolecule at different irradiation times. Fig. 11b shows the accumulated electrons in one supramolecule. From the slope of each step, the formation quantum yields of [(Ring^4+^)–(SiPOM^5−^)]^−^, [(Ring^4+^)–( SiPOM^6−^)]^2−^, and [(Ring^3+^)–(SiPOM^6−^)]^3−^ were obtained as 83%, 32%, and 10%, respectively.

Similar experiments were performed for (Ring^4+^)–(GePOM^4−^), and Fig. S6 and S7† shows the results. In the initial stage, similar spectral changes were observed, that is, a broad absorption appeared until 5 min of irradiation, and then the absorption maxima shifted to 650 nm after irradiation for 20 min. These two spectra can be attributed to the OERS species [(Ring^4+^)–(GePOM^5−^)]^−^ and TWERS species [(Ring^4+^)–(GePOM^6−^)]^2−^ (eqn (10) and (11)) because of their similarities to those of GePOM^5−^ and GePOM^6−^ obtained by flow electrolysis (Fig. 9). Fitting results indicated that the quantum yields of [(Ring^4+^)–(GePOM^5−^)]^−^ and [(Ring^4+^)–(GePOM^6−^)]^2−^ were 85% and 64%, respectively (Fig. S6b†). Further irradiation induced spectral changes from those of (Ring^4+^)–(SiPOM^4−^), that is, an increase in the absorption at *λ*_max_ = ∼360 nm and *λ* > ∼750 nm, and the spectra after irradiation for longer than 30 min can be fitted using the spectra of Ring^4+^, Ring^3+^, GePOM^6−^ and H_2_GePOM^4−^ (Fig. S6c†). The photochemical reduction of [(Ring^4+^)–(GePOM^6−^)]^2−^ should first give the three-electron reduced species [(Ring^3+^)–(GePOM^6−^)]^3−^ (eqn (12)). Because the formation potential of H_2_GePOM^6−^ (*E* = −1.50 V) is similar to that of Ring^3+^ (*E* = −1.54 V), intramolecular electron transfer, which should give [(Ring^4+^)–(GePOM^7−^)]^3−^, and then two molecules of the produced [(Ring^4+^)–(GePOM^7−^)]^3−^ probably underwent disproportionation coupled with protonation, giving [(Ring^4+^)–(H_2_GePOM^6−^)]^2−^, which is a four-electron reduced species, and the TWRS species [(Ring^4+^)–(GePOM^6−^)]^2−^ (eqn (13)).

10 [(Ring^4+^)–(GePOM^4−^)] + e^−^ → [(Ring^4+^)–(GePOM^5−^)]^−^

11 [(Ring^4+^)–(GePOM^5−^)]^−^ + e^−^ → [(Ring^4+^)–(GePOM^6−^)]^2−^

12 [(Ring^4+^)–(GePOM^6−^)]^2−^ + e^−^ → [(Ring^3+^)–(GePOM^6−^)]^3−^

[(Ring^3+^)–(GePOM^6−^)]^3−^ ⇆ [(Ring^4+^)–(GePOM^7−^)]^3−^13
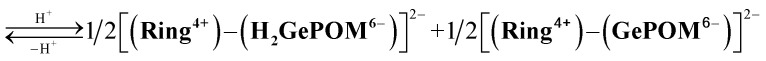


In the DMSO–TEOA (5 : 1 v/v) mixed solution, the emission from (Ring^4+^)–(XPOM^4−^) was almost completely quenched. This effect should be reductive quenching by TEOA: the Stern–Volmer plots in the case of (Ring^4+^)–(SiPOM^4−^) are shown in Fig. S8.† Therefore, there are two photochemical formation routes of OERS [(Ring^4+^)–(XPOM^5−^)]^−^: first, intermolecular reductive quenching of the excited Ring^4+^ unit by TEOA followed by intramolecular electron transfer to the XPOM^5−^ unit (eqn (14)); second, intramolecular electron transfer from the excited Ring^4+^ unit to the XPOM^5−^ unit, followed by reduction of the oxidised ring unit (Ring^5+^) by TEOA (eqn (15)). Although we could not determine the ratio between these two formation processes of [(Ring^4+^)–(XPOM^5−^)]^−^ owing to the complexity of the emission behaviours of excited (Ring^4+^)–(XPOM^4−^), both excited (Ring^4+^)–(SiPOM^4−^) and (Ring^4+^)–(GePOM^4−^) were quantitatively quenched, and the corresponding OERS species formed in good quantum yields, as described above.

In contrast, the second reduction of TWERS, [(Ring^4+^)–(XPOM^6−^)]^2−^, should be produced *via* intermolecular reductive quenching of the excited Ring^4+^ unit in [(Ring^4+^)–(XPOM^5−^)]^−^ by TEOA, followed by intramolecular electron transfer from the Ring^3+^ unit to the XPOM^5−^ unit (eqn (16)), because the reduction potential of XPOM^5−^ (*E*_red_^1/2^ = −1.40 V) is much more negative than the oxidation potential of the ^3^MLCT excited state of Ring^4+^ (
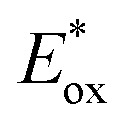
 = −1.0 ∼ −1.1 V). Intramolecular oxidative quenching is a highly endothermic process.14
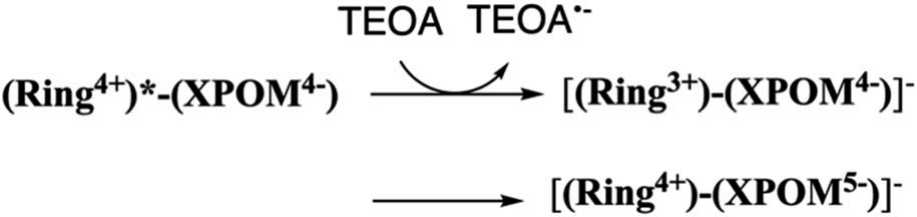
15
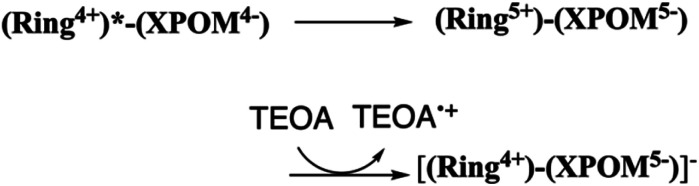
16
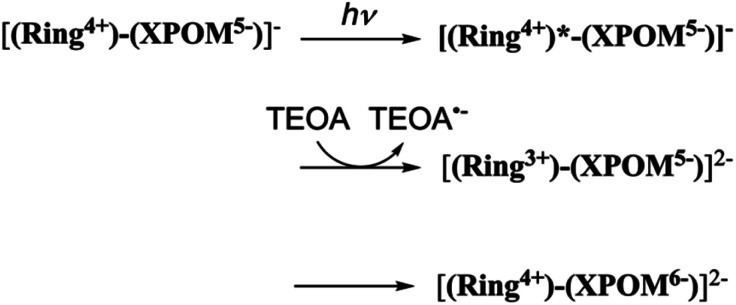


The third reduction process to form [(Ring^5+^)–(SiPOM^6−^)]^3−^ and [(Ring^4+^)–(H_2_GePOM^6−^)]^2−^ should proceed *via* intermolecular reductive quenching of the excited Ring^4+^ unit by TEOA for a similar reason. The quantum yields of the second and third reduction processes were lower than those of the first one. This result is reasonable because the intramolecular electron transfer from the reduced POM unit to the excited Ring^4+^ unit, which is an energy-consuming process owing to the subsequent back-electron transfer, should compete with the intermolecular electron transfer from TEOA.

### Photocatalytic reduction of CO_2_ with RuCAT

**Fig. 12:**
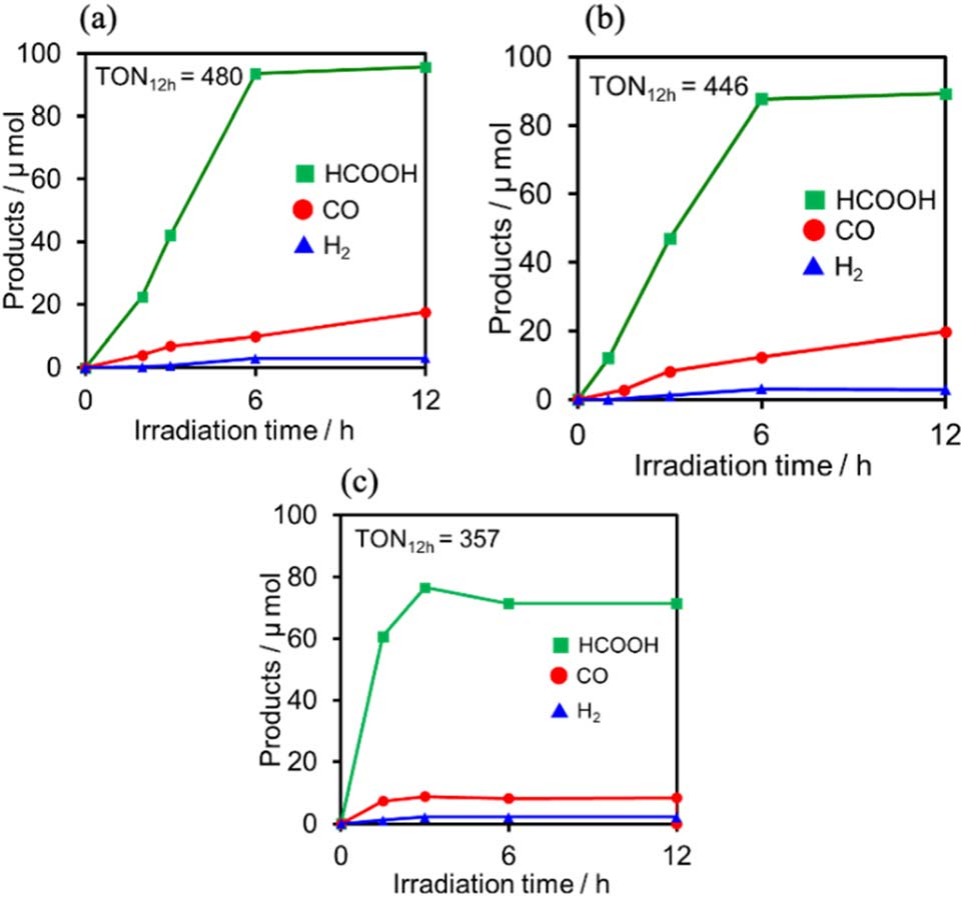
Formation of products during irradiation to DMSO–TEOA (5 : 1 v/v) solutions containing (a) (Ring^4+^)–(SiPOM^4−^) (0.05 mM) and RuCAT (0.05 mM) and (b) (Ring^4+^)–(GePOM^4−^) (0.05 mM) and RuCAT (0.05 mM) and (c) Ring^4+^ (0.05 mM) and RuCAT (0.05 mM) at *λ*_ex_ = 436 nm (2.5 × 10^−7^ einstein s^−1^) under CO_2_ atmosphere.

Photocatalytic CO_2_ reduction was conducted using (Ring^4+^)–(XPOM^4−^) or Ring^4+^ as PS together with RuCAT, which has been frequently used as a catalyst for CO_2_ reduction in photocatalytic and electrocatalytic systems.^7,42,43^ A DMSO–TEOA (5 : 1 v/v) solution containing one of the PSs and RuCAT (0.05 mM each) was irradiated at *λ*_ex_ = 436 nm, with a high light intensity of 2.5 × 10^−7^ einstein s^−1^ under a CO_2_ atmosphere, giving HCOOH as the main product with CO and H_2_ as minor products in all cases (Fig. 12). In the reaction using (Ring^4+^)–(SiPOM^4−^) as PS, HCOOH continuously formed for up to 6 h, and the turnover number and the selectivity of the HCOOH formation were TON_HCOOH_ = 480 and *S*_HCOOH_ = 86%, respectively (Fig. 12a). In the photocatalytic reaction using (Ring^4+^)–(GePOM^4−^) as PS, similar results were obtained; TON_HCOOH_ = 446 (Fig. 12b). Notably, the use of Ring^4+^, a well-known PS as described in the Introduction section, instead of (Ring^4+^)–(XPOM^4−^) induced the lowest durability of photocatalysis (Fig. 12c, TON_HCOOH_ = 357), although the formation speed of HCOOH was faster in the initial stage compared to that of (Ring^4+^)–(XPOM^4−^) as PS. The slower formation of HCOOH in the cases using (Ring^4+^)–(XPOM^4−^) could be caused by the slower reduction processes of their TWERS ([(Ring^4+^)–(XPOM^6−^)]^2−^), compared to that of free Ring^4+^.

The CV of RuCAT measured in a DMSO–TEOA solution containing (TBA^+^)(PF_6_^−^) under a CO_2_ atmosphere showed a broad irreversible wave at *E*_p_ = −1.42 V *vs.* Ag/AgNO_3_ (Fig. 7). Therefore, although the OERS of the Ring^4+^ unit (Ring^3+^) and protonated four-electron reduced species of the GePOM^4−^ (H_2_GePOM^6−^) unit can transfer an electron to RuCAT, the other reduced species of the SiPOM^4−^ and GePOM^4−^ units cannot perform electron transfer to RuCAT because of their more positive redox potentials (Table 2).^40^

**Fig. 13:**
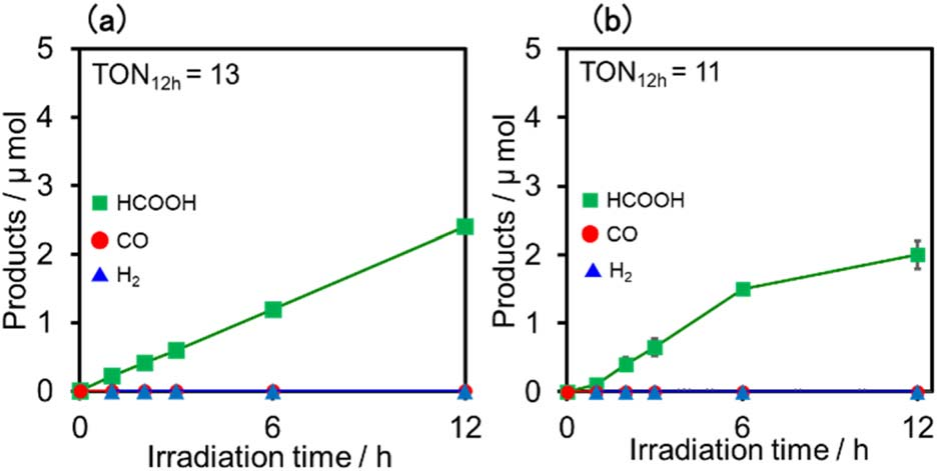
Formation of products during irradiation to DMSO–TEOA (5 : 1 v/v) solutions containing a photosensitiser (0.05 mM) and RuCAT (0.05 mM) at *λ*_ex_ = 436 nm (5.0 × 10^−9^ einstein s^−1^) under CO_2_ atmosphere: the photosensitiser was (a) (Ring^4+^)–(SiPOM^4−^) and (b) (Ring^4+^)–(GePOM^4−^).

Upon irradiation with high light intensity, certain amounts of various species with different reducing powers are accumulated in the photocatalytic reactions, and we cannot separately investigate the roles of each reduced species of the supramolecular photocatalysts (ESI†^41–43^). Therefore, the photocatalytic reactions were conducted using a lower light intensity (5.0 × 10^−9^ einstein s^−1^) to investigate more details of the photocatalytic systems using either (Ring^4+^)–(SiPOM^4−^) or (Ring^4+^)–(GePOM^4−^) as PS by observing the exact amounts of the reduced species of the photosensitisers, as described below. HCOOH was photocatalytically produced as the main product in both cases (Fig. 13).

Similar photoreactions without PS or RuCAT produced only small amounts of HCOOH (Table 3). These results indicate that (Ring^4+^)–(XPOM^4−^) and RuCAT functioned as PS and catalyst, respectively. Using (Ring^4+^)–(SiPOM^4−^) as PS, HCOOH continuously formed even after irradiation for 12 h, and the turnover number and the selectivity of the HCOOH formation were TON_HCOOH_ = 12 and *S*_HCOOH_ = 97% after irradiation for 12 h (Fig. 13a). Although in the photocatalytic reaction using (Ring^4+^)–(GePOM^4−^) as PS, HCOOH was also produced as the main product, an induction period was observed in the initial stage, and the formation rate of HCOOH slowed down after irradiation for 6 h (Fig. 13b), in which TON_HCOOH_ was 11 after 12 h irradiation (*S*_HCOOH_ = 94%).^44^

**Table 3:** Photocatalytic reactions using **RuCAT**^a^

Photosensitiser	Catalyst	Time	Products/μmol		TON_HCOOH_
		h	HCOOH	CO	
(Ring^4+^)–(SiPOM^4−^)	RuCAT	1	0.2	Trace	1
		6	1.2	Trace	6
		12	2.4	Trace	12
(Ring^4+^)–(GePOM^4−^)	RuCAT	1	0.1	Trace	0.5
		6	1.5	Trace	7.5
		12	2.0	Trace	10
(Ring^4+^)–(SiPOM^4−^)	—b	12	Trace	Trace	
(Ring^4+^)–(GePOM^4−^)	—b	12	Trace	Trace	
—c	RuCAT	12	Trace	Trace	

a DMSO–TEOA (5 : 1 v/v) solution containing PS (0.05 mM) and/or RuCAT (0.05 mM) was irradiated at *λ*_ex_ = 436 nm (5.0 × 10^−9^ einstein s^−1^) under a CO_2_ atmosphere.

b In the absence of RuCAT.

c In the absence of (Ring^4+^)–(XPOM^4−^).

**Fig. 14:**
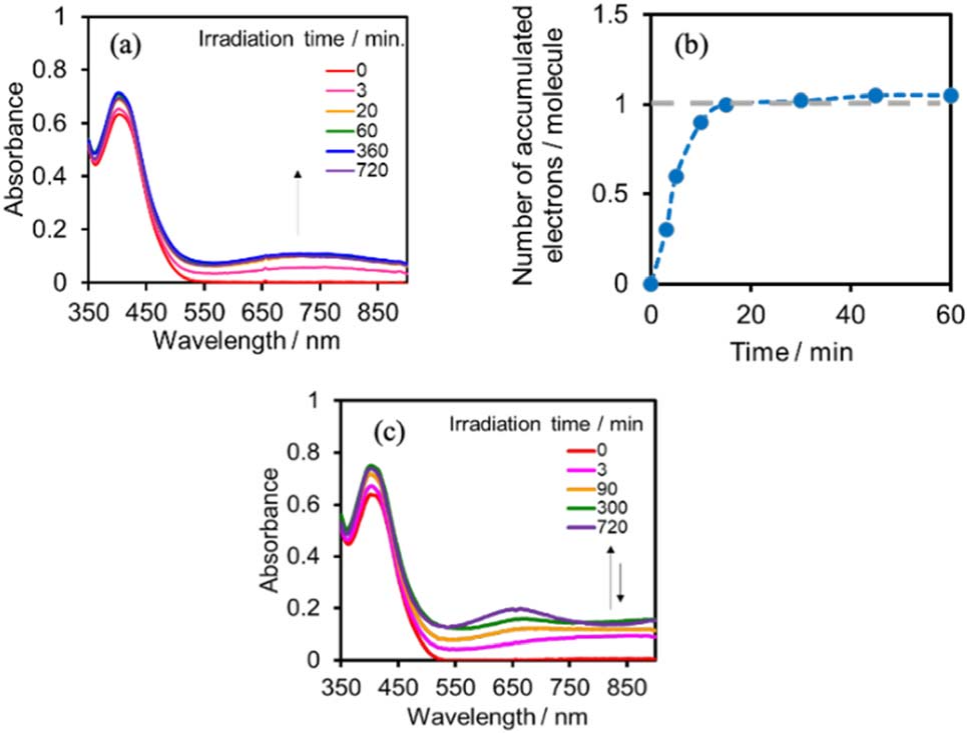
(a) UV-vis absorption spectra of the DMSO–TEOA (5 : 1 v/v) solution containing (Ring^4+^)–(SiPOM^4−^) (0.05 mM) and RuCAT (0.05 mM) during irradiation at *λ*_ex_ = 436 nm (5.0 × 10^−9^ einstein s^−1^) under a CO_2_ atmosphere, and (b) accumulated electrons in one molecule of (Ring^4+^)–(SiPOM^4−^). (c) UV-vis absorption spectra of the DMSO–TEOA (5 : 1 v/v) solution containing (Ring^4+^)–(GePOM^4−^) (0.05 mM) and RuCAT (0.05 mM) during irradiation at *λ*_ex_ = 436 nm (5.0 × 10^−9^ einstein s^−1^) under a CO_2_ atmosphere.

We investigated the electron-accumulation behaviour of (Ring^4+^)–(XPOM^4−^) during photocatalytic reactions with low light intensity (5.0 × 10^−9^ einstein s^−1^). Fig. 14a shows the UV-vis absorption spectra of the reaction solution with (Ring^4+^)–(SiPOM^4−^) as the PS during irradiation. In the initial stage, OERS ([(Ring^4+^)–(SiPOM^5−^)]^−^) was formed with a similar time scale to the photoreaction without RuCAT (Fig. 10a). The formation yield of the OERS was ∼100% after 18 min of irradiation (Fig. 14b). Although longer irradiation times slowly induced the formation of a small amount of TWERS ([(Ring^4+^)–(SiPOM^6−^)]^2−^), the yield did not change for up to 12 h. In other words, most of the produced TWERS were consumed during the photocatalytic reaction. Three-electron reduced species, that is, [(Ring^3+^)–(SiPOM^6−^)]^3−^ was not observed at all. Based on these results and the similar formation potentials of the TWERS and the OERS of RuCAT (Table 2), we can conclude that the TWERS can slowly supply one electron to RuCAT (eqn (17)) and can efficiently supply another electron to intermediate(s) as well, which should be produced from the OERS of RuCAT and CO_2_ and/or H^+^, giving HCOOH (eqn (18) and (19)). This second supply of electrons from the TWERS should be sufficiently fast to suppress the decomposition of the Ru catalyst, which should be polymerization of reduced Ru complexes^41–43^ (eqn (20), ESI†), and recover the OERS. In other words, TWERS (*E*_1/2_red = −1.40 V) has sufficient reduction potential to reduce the intermediate(s) of the formation of HCOOH with high efficiency.17
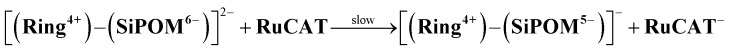
18
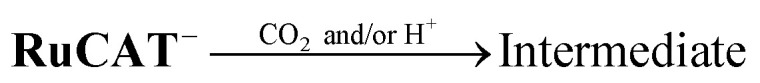
19
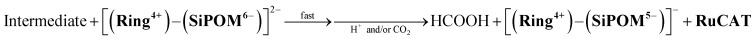
20
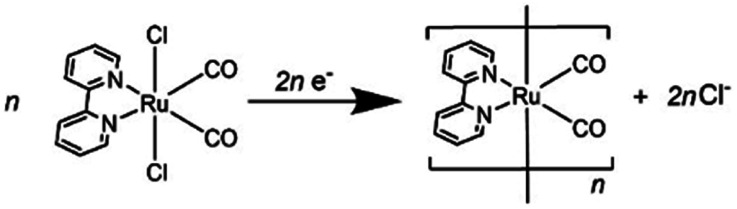


Notably, at higher light intensities (2.5 × 10^−7^ einstein s^−1^), a considerable amount of TWERS [(Ring^4+^)–(SiPOM^6−^)]^2−^ accumulated in the reaction solution (Fig. 10b), owing to the much higher photochemical electron supply from the Ring^4+^ unit. Under such conditions, the three-electron reduced species [(Ring^3+^)–(SiPOM^6−^)]^3−^ is photochemically produced during the photocatalytic reaction and supplies an electron to RuCAT (eqn (21)). This electron supply should be much faster than that from the TWERS because of the much higher reduction potential of [(Ring^3+^)–(SiPOM^6−^)]^3−^ (Table 2).21
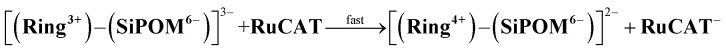


Fig. 14c shows the UV-vis absorption spectral changes of the solution in the case of (Ring^4+^)–(GePOM^4−^) during the photocatalytic reaction using a low light intensity (5.0 × 10^−9^ einstein s^−1^) for 60 min. Accumulation of OERS [(Ring^4+^)–(GePOM^5−^)]^−^ was also observed in this photocatalytic reaction solution during irradiation. After irradiation for 10 min, one-electron reduction of nearly all of (Ring^4+^)–(GePOM^4−^) proceeded, in which the production speed of the OERS was slightly faster than that using (Ring^4+^)–(SiPOM^4−^) as PS. Further irradiation induced evident spectral changes; the accumulation of TWERS ([(Ring^4+^)–(GePOM^6−^)]^2−^) showed an absorption maximum at ∼650 nm. In addition, another species with an absorption band at longer wavelengths suggested the formation of a Ru polymer^41–43^ (eqn (20), ESI†).

**Fig. 15:**
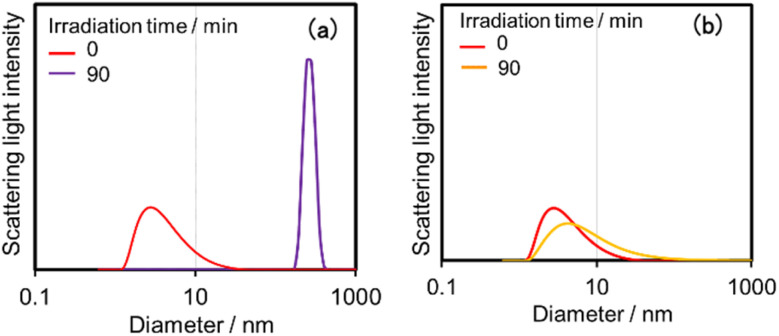
Particle size distributions of the photocatalytic reaction solutions before and after irradiation for 90 min in the case using (Ring^4+^)–(GePOM^4−^) (a) or (Ring^4+^)–(SiPOM^4−^) (b) as PS. The DMSO–TEOA (5 : 1 v/v) solutions containing PS (0.05 mM) and RuCAT (0.05 mM) were irradiated at *l*_ex_ = 436 nm under a CO_2_ atmosphere.

DLS was applied to the photocatalytic reaction solutions (irradiated for 90 min) to detect the Ru polymer. In the case of (Ring^4+^)–(GePOM^4−^), not only particles with *D* = several nanometres, which are attributed to (Ring^4+^)–(GePOM^4−^) and partially to the accumulated oligomers, but also much larger particles with *D* = 140 nm, which are attributable to the Ru polymer (Fig. 15a). However, when using (Ring^4+^)–(SiPOM^4−^), such large particles were not observed after the photocatalytic reaction (Fig. 15b). Therefore, under these reaction conditions (low light intensity), Ru polymer formation was suppressed during the photocatalytic reaction using (Ring^4+^)–(SiPOM^4−^) as the PS but not entirely in the system using (Ring^4+^)–(GePOM^4−^).

These results indicate that in the photocatalytic system using (Ring^4+^)–(GePOM^4−^) as the PS, the TWERS [(Ring^4+^)–(GePOM^6−^)]^2−^ supplies an electron to RuCAT at a slower rate than the system using (Ring^4+^)–(SiPOM^4−^) owing to its lower reduction ability (Table 2). The second electron donation from the TWERS ([(Ring^4+^)–(GePOM^6−^)]^2−^) to the intermediate produced from the OERS of RuCAT should also be slower than that from [(Ring^4+^)–(SiPOM^6−^)]^2−^ because of the faster decline in photocatalysis and the formation of the Ru polymer, even in the presence of TWERS. This provides information on the redox properties of the reaction intermediate for photocatalytic CO_2_ reduction using RuCAT. Since the intermediate can be efficiently reduced by the TWERS of [(Ring^4+^)–(SiPOM^4−^)] ([(Ring^4+^)–(SiPOM^6−^)]^2−^) of which oxidation potential is *E*_1/2_ = −1.40 V, the reduction potential of the intermediate should be more positive than −1.40 V. In contrast, in the case using (Ring^4+^)–(GePOM^4−^) as PS, reduction of the intermediate by the TWERS ([(Ring^4+^)–(GePOM^6−^)]^2−^) was slow. In other words, the reduction potential of the intermediate should be similar to or slightly more negative than the redox potential of the [(Ring^4+^)–(GePOM^6−^)]^2−^/[(Ring^4+^)–(GePOM^5−^)]^−^ couple, that is, *E*_2/1_ = −1.33 V. Notably, determining the reduction potential of the reaction intermediate is difficult in principle when the reduction potential of the starting catalyst is more negative than that of the short-lived intermediate, as described in the Introduction section.

### Photocatalytic reduction of CO_2_ with ReCAT

*fac-*[Re^I^(N^N)(CO_3_)L]^*n*+^-type complexes are probably the most frequently used catalysts in both photocatalytic and electrocatalytic systems for CO_2_ reduction.^7^ In particular, *fac-*[Re(bpy)(CO_3_){OC(O)OC_2_H_4_N(C_2_H_4_OH)_2_}] (ReCAT) incorporates CO_2_ as a carbonate ester ligand with a deprotonated TEOA unit. This compound has been reported to function as an excellent catalyst for CO_2_ reduction in photocatalytic^45^ and electrocatalytic^46^ systems. The CV of ReCAT measured in a DMSO–TEOA solution containing (TBA^+^)(PF_6_^−^) under a CO_2_ atmosphere showed a broad irreversible wave at Ep^red^ = −1.50 V *vs.* Ag/AgNO_3_ (Fig. 7). Therefore, we can expect that the OERS of Ring^4+^ (Ring^3+^) and the (protonated) four-electron reduced species of GePOM^4−^ (H_2_GePOM^6−^) should be able to transfer an electron to ReCAT while the OERS and TWERS of SiPOM^4−^ and GePOM^4−^ are unable to do so because of their more positive redox potentials (Fig. 7, Table 2).

**Fig. 16:**
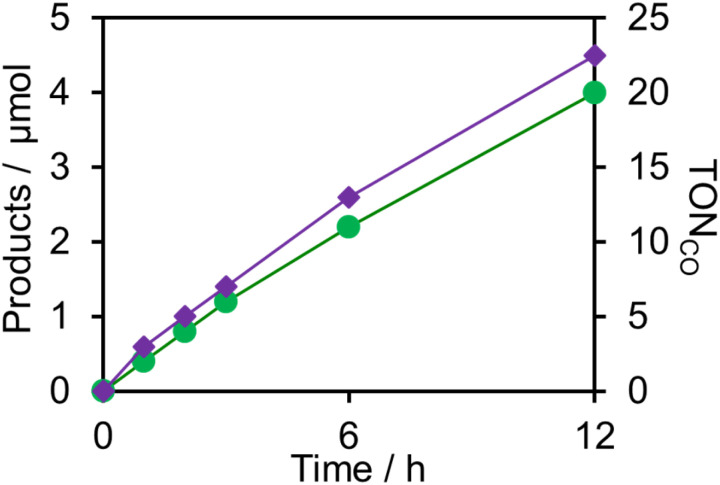
Photocatalytic CO_2_ reduction using (Ring^4+^)–(XPOM^4−^) (0.05 mM) and ReCAT (0.05 mM) in DMSO–TEOA (5 : 1 v/v) solution (*λ*_ex_ = 436 nm: 5.0 × 10^−9^ einstein s^−1^); green (Ring^4+^)–(SiPOM^4−^), purple (Ring^4+^)–(GePOM^4−^).

A DMSO–TEOA (5 : 1 v/v) solution containing (Ring^4+^)–(SiPOM^4−^) and ReCAT (0.05 mM each) was irradiated at *λ*_ex_ = 436 nm with a low light intensity (5.0 × 10^−9^ einstein s^−1^) under a CO_2_ atmosphere, giving CO selectively and continuously for 12 h (TON_CO_ = 20, *S*_CO_ > 99%) (Fig. 16). A similar reaction, except when using (Ring^4+^)–(GePOM^4−^) as PS instead of (Ring^4+^)–(SiPOM^4−^), gave similar results (TON_CO_ = 23, *S*_CO_ > 99%). The quantum yields of CO formation were approximately 2% in each case using (Ring^4+^)–(SiPOM^4−^) or (Ring^4+^)–(SiPOM^4−^) as PS and ReCAT as the catalyst.^47^

**Fig. 17:**
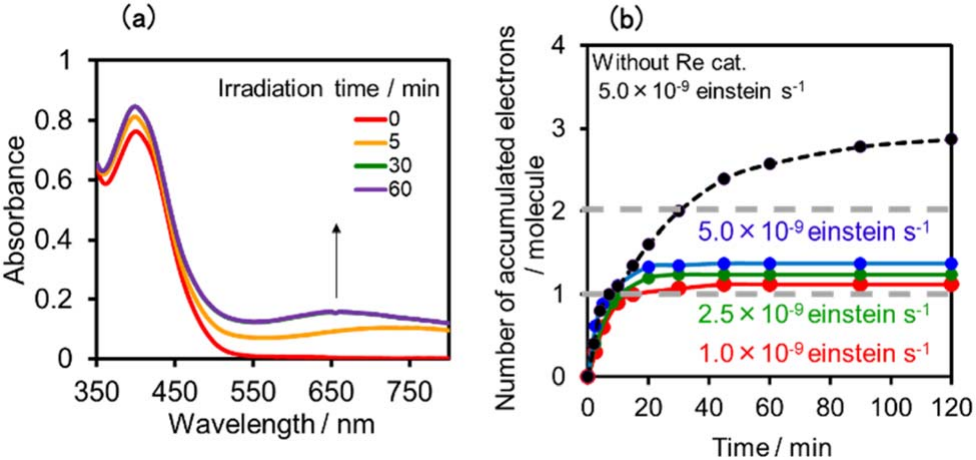
(a) UV-vis absorption spectra of the DMSO–TEOA (5 : 1 v/v) solution containing (Ring^4+^)–(SiPOM^4−^) (0.05 mM) and ReCAT(0.05 mM) during irradiation at *λ*_ex_ = 436 nm under a CO_2_ atmosphere, and (b) accumulated electrons in one molecule of (Ring^4+^)–(SiPOM^4−^).

Fig. 17a shows the changes in UV-vis absorption during the photocatalytic reaction using (Ring^4+^)–(SiPOM^4−^) as the PS. In the initial stage (irradiation time < 5 min), the OERS ([(Ring^4+^)–(SiPOM^5−^)]^−^) accumulated, similar to the photoreaction in the absence of ReCAT (Fig. 10a). The following processes were different in the presence and absence of ReCAT, that is, the accumulated amount of the TWERS ([(Ring^4+^)–(SiPOM^5−^)]^2−^) was lower, and the three-electron reduction species ([(Ring^3+^)–(SiPOM^6−^)]^3−^) was not observed in the presence of the catalyst. The yield of TWERS reached a maximum (40%) after 20 min irradiation, and 60% of the OERS remained in the solution. The yield of the accumulated TWERS was dependent on the irradiation light intensity; a lower light intensity induced a lower yield (Fig. 17b). The maximum yield of [(Ring^4+^)–(SiPOM^5−^)]^2−^ was only 10% when the light intensity was 1.0 × 10^−9^ einstein s^−1^. Notably, the photocatalytic CO formation proceeded at this low light intensity (TON_CO_ = 12 after 12 h of irradiation). Based on these results, we deduced that two electrons were donated from the three-electron reduced species [(Ring^3+^)–(SiPOM^6−^)]^3−^ during photocatalytic CO_2_ reduction using (Ring^4+^)–(SiPOM^4−^) as the PS and ReCAT as the catalyst. The first electron transfer should proceed from [(Ring^3+^)–(SiPOM^6−^)]^3−^ to ReCAT to give the TWERS and OERS of ReCAT (ReCAT^−^) because electron transfer from the TWERS to ReCAT was endergonic. The produced TWERS should provide one more electron to an intermediate produced from ReCAT^−^ because TWERS is consumed during the photocatalytic reaction. Therefore, the intermediate can be reduced by the species with *E*_1/2_ = −1.40 V, but this second electron transfer was slower than that in the system using RuCAT and (Ring^4+^)–(SiPOM^4−^). Therefore, the reduction potential of the intermediate obtained from the OERS of ReCAT is close to −1.40 V.

**Fig. 18:**
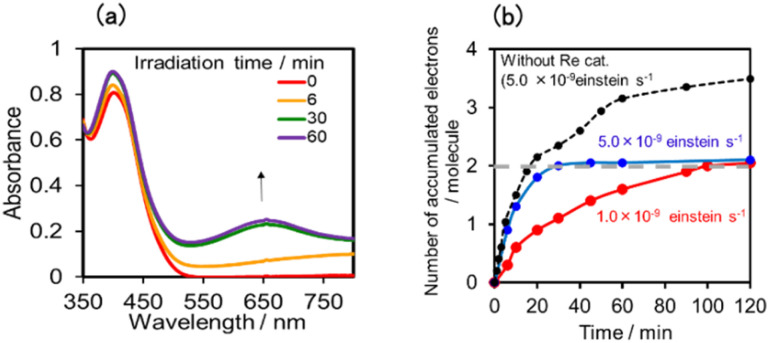
(a) UV-vis absorption spectra of the DMSO–TEOA (5 : 1 v/v) solution containing (Ring^4+^)–(GePOM^4−^) (0.05 mM) and ReCAT(0.05 mM) during irradiation at *λ*_ex_ = 436 nm under a CO_2_ atmosphere, and (b) accumulated electrons in one molecule of (Ring^4+^)–(GePOM^4−^).

In the photocatalytic reaction using (Ring^4+^)–(GePOM^4−^) as PS and ReCAT as a catalyst, all (Ring^4+^)–(GePOM^4−^) were converted into the TWERS ([(Ring^4+^)–(GePOM^6−^)]^2−^); that is, the TWERS were fully accumulated not only with an irradiation light intensity of 5.0 × 10^−9^ einstein s^−1^ (*λ*_ex_ = 436 nm) but also with a lower intensity (1.0 × 10^−9^ einstein s^−1^) (Fig. 18a and b). This result indicates that the intermediate produced from the OERS of ReCAT cannot be reduced by TWERS [(Ring^4+^)–(GePOM^6−^)]^2−^, whose redox potential is *E*_1/2_ = −1.33 V. This result is consistent with the results obtained using (Ring^4+^)–(SiPOM^4−^) as the reduction of the intermediate proceeded by [(Ring^4+^)–(SiPOM^6−^)]^2−^, with a redox potential of *E*_1/2_ = −1.40 V, only at a slow rate. Therefore, in this photocatalytic reaction using (Ring^4+^)–(GePOM^4−^) as the PS, the intermediate must also be reduced only by [(Ring^4+^)–(H_2_GePOM^6−^)]^2−^ (and partially by an α-amino radical produced by deprotonation of the oxidised TEOA^40^). Notably, the formation rate of [(Ring^4+^)–(H_2_GePOM^6−^)]^2−^ was slightly higher than that of [(Ring^3+^)–(SiPOM^6−^)]^3−^ under these reaction conditions (Fig. 11a and S7a). This effect is probably the reason for the similar CO formation rates in the two photocatalytic systems. Based on the results and the investigations, we can deduce that, in the photocatalytic reduction of CO_2_ using ReCAT, the reduction potential of the intermediate produced from ReCAT^−^ is −1.33V < *E* ≤ −1.40 V. We recently clarified that the following process of ReCAT^−^ to the intermediate is a unimolecular reaction with a rate constant of *k* = 1.8 s^−1^ at 298 K.^48^ Although several assumptions of the structure of the intermediate were reported, this is the first information about a redox potential of the intermediate in the photocatalytic reactions using Re(i)-complex catalysts to the best of our knowledge.

This method for determining the reduction potentials of the reaction intermediates was applied to another Re(i) complex, *fac-*[Re(Me_2_bpy)(CO_3_){OC(O)OC_2_H_4_N(C_2_H_4_OH)_2_}] (ReMeCAT, Me_2_bpy = 4,4′-dimethyl-2,2′-bipyridine, Fig. S10†), which has a more negative reduction potential (Ep^red^ = −1.64 V, Fig. S11†) than that of ReCAT (Ep^red^ = − 1.50 V). We can expect the intermediate produced from the OERS of ReMeCAT to have a more negative reduction potential than that of ReCAT if the intermediates have similar structures. In this case, the RingMe^4+^ unit with Me_2_bpy ligands instead of the bpy ligands must be used instead of the Ring^4+^ unit in the PS because the reduction power of Ring^3+^ (*E*_1/2_(Ring^4+/3+^) = −1.54 V) is not sufficient to reduce ReMeCAT. In contrast, RingMe^3+^ (*E*_1/2_(RingMe^4+/3+^) = − 1.78 V, Fig. S11†) can reduce ReMeCAT. A DMSO–TEOA (5 : 1 v/v) solution containing (RingMe^4+^)–(SiPOM^4−^) and ReMeCAT (0.05 mM each) was irradiated at *λ*_ex_ = 436 nm with a low light intensity (5.0 × 10^−9^ einstein s^−1^) under a CO_2_ atmosphere, also giving CO selectively (TON_CO_ = 43 after irradiation for 12 h, Table S4†). The UV-vis absorption changes of the photocatalytic reaction solution indicate that two electrons were accumulated in (RingMe^4+^)–(SiPOM^4−^) in the photostationary state of the photocatalytic reaction (Fig. S12;† the details of these experimental results are described in the ESI†). This clearly shows that TWERS [(RingMe^4+^)–(SiPOM^6−^)]^2−^ cannot reduce the intermediate produced from the OERS of ReMeCAT. Therefore, we can conclude that the intermediate has a more negative reduction potential than −1.40 V. These results strongly support the reliability of this estimation method for the reduction potential of the reaction intermediate.

## Conclusions

We synthesised supramolecular photosensitisers consisting of ring-shaped tetranuclear Re(i) complexes and Keggin-type heteropolyoxometalates in a 1 : 1 ratio. These supramolecular photosensitisers can photochemically accumulate multi-electrons in the presence of TEOA as the electron donor. In some combinations of photosensitisers and catalysts, supramolecular photosensitisers can donate two electrons to the catalyst and the intermediate to induce the photocatalytic reduction of CO_2_ to CO or HCOOH. In molecular photocatalytic systems, CO_2_ reduction generally proceeds *via* an intermediate produced by the OERS of the catalyst and CO_2_ and/or H^+^. In many cases, since the reduction potential of the intermediate is more positive than the first reduction potential of the catalyst, it is a challenge to obtain information on the redox properties of the intermediate. Moreover, electrons accumulated in supramolecular photosensitisers have different reduction powers; therefore, we can use these supramolecular photosensitisers to gain insight into the reduction potentials of the intermediates produced from the OERSs of the catalysts. The following conclusions were drawn from this study:

(i) The reduction of the intermediate from the OERS of ReCAT does not proceed using an electron donor (reduced PS) with *E*^red^_1/2_ = −1.33 V but proceeds with *E*^red^_1/2_ = −1.40 V.

(ii) The intermediate from the OERS of ReMeCAT cannot be reduced, even by the reduced PS with *E*^red^_1/2_ = −1.40 V.

(iii) The reduction of the intermediate made from the OERS of RuCAT and CO_2_ barely proceeded with the reduced PS with *E*^red^_1/2_ = −1.33 V.

These photochemical methods for supplying multi-electrons with different potentials from one molecule should also aid in clarifying the redox properties of the intermediates of other photocatalytic reactions, such as H_2_ evolution and photoredox catalytic reactions, providing multi-electron reduction products. In addition, the supramolecular photosensitizers developed in this study can suppress decomposition of intermediates produced from OERS of the catalyst owing to their rapid second electron donating abilities to increase durability of the photocatalysis.

## Data availability

Data supporting the work within this manuscript is included within the full text of the manuscript and the provided ESI.†

## Author contributions

MT and TA did most of the experiments and equally contributed. TM and YT guided them. NH made crystals of the samples. YK measured the X-ray diffraction data and analyzed the crystal structures, and KF and MY supported YK. OI conceived and leaded this research, and leaded to write this paper.

## Conflicts of interest

There are no conflicts to declare.

## Supplementary Material

SC-014-D2SC04252E-s001

SC-014-D2SC04252E-s002
