# Supplementary material for: Supramolecular multi-electron redox photosensitisers comprising a ring-shaped Re(i) tetranuclear complex and a polyoxometalate
Source: Chem Sci. 2022 Dec 1;14(3):691–704. doi: 10.1039/d2sc04252e (PMC9848162; doi:10.1039/d2sc04252e)
Supplement: SC-014-D2SC04252E-s001 [file SC-014-D2SC04252E-s001.pdf]

Electronic Supplementary Material (ESI) for Chemical Science.
This journal is © The Royal Society of Chemistry 2022

SUPPORTING INFORMATION

Supramolecular Multi-Electron Redox Photosensitizers Consisting of a Ring-Shaped Re(I) Tetranuclear Complex and a Polyoxometalate

Maria Takahashi,[1] Tsuyoshi Asatani,[1] Tatsuki Morimoto,[2] Yoshinobu Kamakura,[1] Kotaro Fujii,[1] Masatomo Yashima,[1] Naoki Hosokawa,[1] Yusuke Tamaki,[1] and Osamu Ishitani[1,3]*

[1]Department of Chemistry, School of Science, Tokyo Institute of Technology, O-okayama 2-12-1-NE-1, Meguro-ku, Tokyo 152-8550, Japan

[2]School of Engineering, Tokyo University of Technology, 1404-1 Katakura, Hachioji, Tokyo 192-0982, Japan

[3]Department of Chemistry, Graduate School of Advanced Science and Engineering, Hiroshima University, 1-3-1 Kagamiyama, Higashi-Hiroshima, Hiroshima 739 8526, Japan

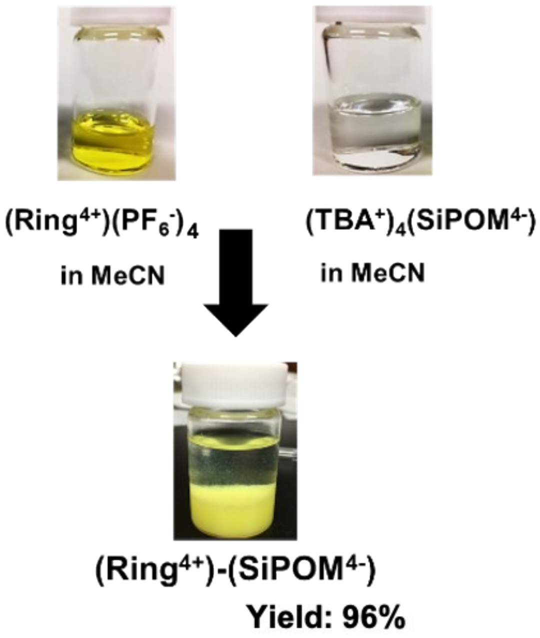

Figure S1. Synthesis of **($Ring^{4+}$)-($SiPOM^{4-}$)**

Table S1. Crystallographic data of (**$Ring^{4+}$)-($SiPOM^{4-}$**) and (**$Ring^{4+}$**)(PF_6)$_4$.

Compound	**($Ring^{4+}$)-($SiPOM^{4-}$)**	(**$Ring^{4+}$**)(PF_6)$_4$
Temperature / K	123	123
Crystal system	triclinic	monoclinic
Space group	*P*-1	*C*2/c
a/Å	18.8254(3)	29.0838(11)
b/Å	23.1045(3)	16.4902(8)
c/Å	33.0343(4)	38.9836(11)
α/°	101.3070(10)	90
β/°	101.0640(10)	94.942(3)
γ/°	112.6480(10)	90
Volume/Å^3	12422.4(3)	18626.9(13)

Z	2	8
ρ_{calc}/g cm^{-3}	1.681	1.320
μ/mm^{-1}	7.582	2.739
F(000)	5860	7384
Crystal size/nm^3	0.13, 0.07, 0.02	0.03, 0.03, 0.01
Wavelength/Å	0.71073	0.71073
θ range for data collection/^{o}	2.4000 to 30.4120	2.4020 to 26.1570
Index ranges	$-22 \le h \le 22$	$-33 \le h \le 34$
	$-27 \le k \le 27$	$-19 \le k \le 19$
	$-39 \le l \le 39$	$-46 \le l \le 46$
Goodness-of-Fit on F^2	1.080	1.073
Final R_1 index [I≥2σ (I)]	0.0498	0.0894
Final R_1 index [all data]	0.0629	0.1410
Final wR_2 index [I≥2σ (I)]	0.1463	0.2470
Final wR_2 index [all data]	0.1533	0.2719

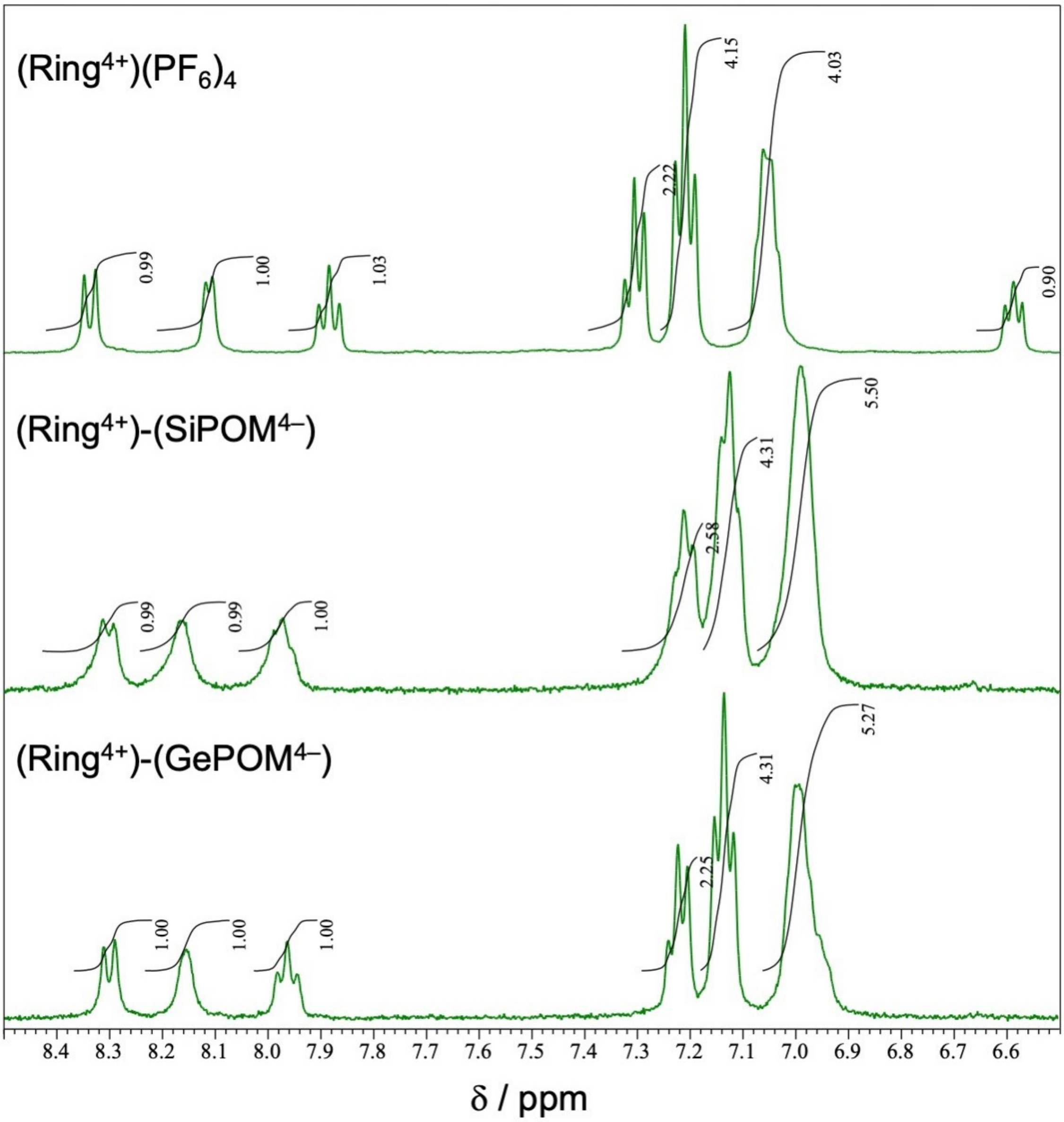

Figure S2. ^{1}H-NMR spectra of (**Ring^{4+}**)(PF_6)$_4$, **(Ring^{4+})-(SiPOM^{4-})**, **(Ring^{4+})-(GePOM^{4-})** and in DMSO-d_6.

Table S2. ^{1}H-NMR data of (**Ring^{4+}**)(PF_6)$_4$, **(Ring^{4+})-(SiPOM^{4-})**, and (**Ring^{4+}**)-(**GePOM^{4-}**).

Complex	Chemical shift / ppm						
	bpy-4	Ph-2	Ph-3	Ph-4	bpy-5	bpy-3	bpy-6
(**Ring^{4+}**)(PF_6)$_4$	6.59	7.05	7.20	7.30	7.88	8.11	8.33
**(Ring^{4+})-**	~7^{b}	7.0 ^{a}	7.13	7.21	7.97	8.17^{a}	8.32

**($SiPOM^{4-}$)**							
**($Ring^{4+}$)-($GePOM^{4-}$)**	~7[b]	7.0 [a]	7.14	7.22	7.96	8.15[a]	8.30

[a] Highest position of the broad peak. [b] This peak is covered by the peak of the Ph-2.

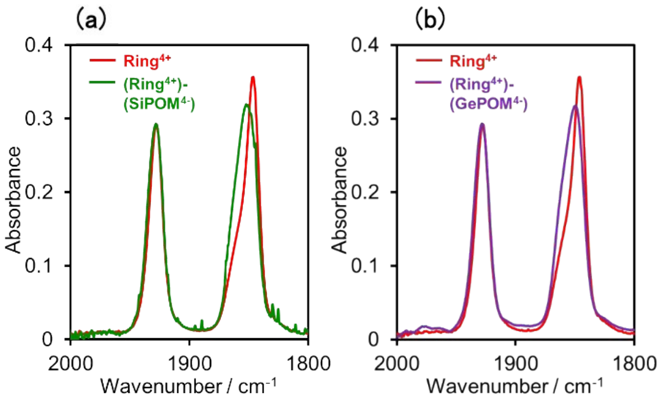

Figure S3. FT-IR spectra of (**$Ring^{4+}$**)(PF_6)$_4$ and (a) (**$Ring^{4+}$)-($SiPOM^{4-}$**) or (b) (**$Ring^{4+}$)-($GePOM^{4-}$**) measured in DMSO solutions.

Detailed investigation of emission decays of Ring^{4+} and (Ring^{4+})-(XPOM^{4-})

Figure S3 shows emission decays of **Ring^{4+}** and **(Ring^{4+})-(XPOM^{4-})** dissolved in the DMSO solutions using the single-photon counting method, normalised by absorbed photon numbers (λ_{ex} = 400 nm, λ_{det} = 615 nm). It was reported that **Ring^{4+}** dissolved in solvent shows multi-emission lifetimes owing to multiple relatively stable conformers, which have different strengths of weak interaction among the bpy ligand and the phenyl groups of the phosphine ligands in solutions.[1,2] In the DMSO solution, the emission decay of the free **Ring^{4+}** could be fitted by using a double exponential function with τ_1 = 406 ns (percentage of pre-exponential factors a_1 = 95%) and τ_2 = 225 ns (a_2 = 5%). In contrast, the fitting of the emission decay of **(Ring^{4+})-(SiPOM^{4-})** required quadruple exponential function, of which two minor components (τ_3 = 372 ns: a_3 = 13%, τ_4 = 188 ns: a_4 = 17%) had similar lifetimes to those of the free **Ring^{4+}**, and lifetimes of the other major components were shorter (τ_5 = 87 ns: a_5 = 39%, τ_6 = 21 ns: a_6 = 31%).

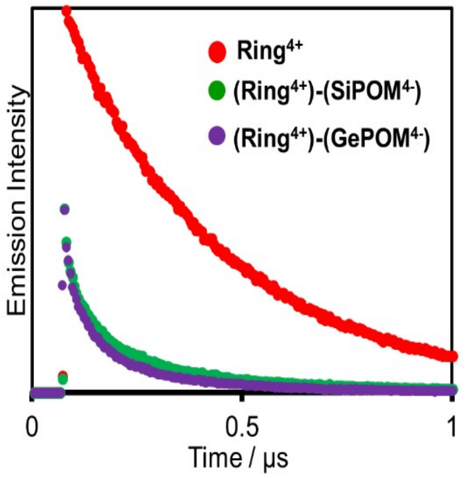

Figure S4. Emission decays of **Ring^{4+}** (red), **(Ring^{4+})-(SiPOM^{4-})** (green), and **(Ring^{4+})-(GePOM^{4-})** (purple): λ_{ex} = 400 nm, λ_{det} = 615 nm, concentrations of the complexes = 0.05 mM: the number of integration was unified.

Notably, the emission strength at time = 0 was very different between **(Ring^{4+})-(SiPOM^{4-})** and free **Ring^{4+}**, i.e., the former was much weaker than the latter when the number of integrations was unified. This result clearly indicates that static quenching of the excited state of the **Ring^{4+}** unit by the **SiPOM^{4-}** unit rapidly proceeded within the time resolution of the apparatus (200 ps). Although the similar lifetimes of the free **Ring^{4+}** indicate that partial ion separation of **(Ring^{4+})-(SiPOM^{4-})** might proceed in the DMSO solution of **(Ring^{4+})-(SiPOM^{4-})**, the effect should be minor because their percentages of pre-exponential factors (a_3 and a_4 in Table S1) were lower than those of the shorter

emission components (a_5 and a_6). If the conformer of free **Ring^{4+}** with a shorter emission lifetime (τ_2 = 225 ns) is dynamically quenched by the total concentration of the **SiPOM^{4-}** unit (0.05 mM) to give an emission lifetime τ_5 = 87 ns, the quenching rate (k_q) can be calculated using Eq. S1.

$$k_q = \left(\frac{1}{\tau_5} - \frac{1}{\tau_2}\right) \div \left(0.05 \times 10^{-3}\right) = 1.4 \times 10^{11} \left[s^{-1}\right] \qquad (S1)$$

Although this value could be attributed to the slowest quenching process of the ^{3}MLCT excited state of free **Ring^{4+}** by **SiPOM^{4-}** in the DMSO solution of **(Ring^{4+})-(SiPOM^{4-})**, this process was much faster than the diffusion-controlled process. Notably, the actual concentration of free **SiPOM^{4-}** should be significantly lower than the added **(Ring^{4+})-(SiPOM^{4-})** (0.05 mM) owing to the formation of supramolecules with **Ring^{4+}** in the solution. These results also indicated that the intramolecular static quenching of the ^{3}MLCT excited state of the **Ring^{4+}** unit by the **SiPOM^{4-}** unit proceeded predominantly and efficiently, and the dynamic quenching of the excited state of the free **Ring^{4+}** and/or **Ring^{4+}** unit by the free **SiPOM^{4-}** and/or **SiPOM^{4-}** units did not proceed or was a minor process. If we can assume that the emission observed in the DMSO solution of **(Ring^{4+})-(SiPOM^{4-})** with similar lifetimes, i.e., τ_3 = 372 ns and τ_4 = 188 ns, was produced by free **Ring^{4+}**, the percentage of dissociation of **(Ring^{4+})-(SiPOM^{4-})** was calculated as only 3.9% using Eq. S2

$$\frac{[\text{free Ring}^{4+}]}{[\text{added }(\text{Ring}^{4+})\text{ - }(\text{SiPOM}^{4-})]} = \frac{\Phi_{\text{em}}((\text{Ring}^{4+})\text{ - }(\text{SiPOM}^{4-}))}{\Phi_{\text{em}}((\text{Ring}^{4+})(\text{PF}_6^{\ -})_4)} \times \frac{a_3 + a_4}{100} = 0.039 \qquad (S2)$$

Therefore, based on the results about emission, we can conclude that more than 85% of the excited state of the **Ring^{4+}** unit was statically quenched by the **SiPOM^{4-}** unit in the DMSO solution dissolving 0.05 mM of **(Ring^{4+})-(SiPOM^{4-})**.

In the DMSO solution containing 0.05 mM of **(Ring^{4+})-(GePOM^{4-})**, a similar emission quenching and shortening of the emission lifetime (Table S2) were observed compared to the free **Ring^{4+}**. These findings show that the supramolecular structure of **(Ring^{4+})-(GePOM^{4-})** was maintained when the **Ring^{4+}** unit was excited. Additionally,

the ^{3}MLCT excited state of the **Ring^{4+}** unit was efficiently quenched by the **GePOM^{4-}** unit in the **(Ring^{4+})-(GePOM^{4-})** as well (more than 87%).

Table S3 Emission lifetimes of the complexes [a]

Materials	τ_n / ns (a_n / %) [b]			
	n = 1	2	3	4
(Ring^{4+})-(SiPOM^{4-})	372 (13)	188 (17)	87 (39)	21 (31)
(Ring^{4+})-(GePOM^{4-})	362 (12)	141 (24)	55 (41)	10 (36)
(**Ring^{4+}**)(PF_6)$_4$	406 (95)	225 (5)		

[a] Measured in DMSO at λ_{ex} = 400 nm and λ_{det} = 615 nm. [b] Percentage of pre-exponential factors.

References

1. T. Morimoto, C. Nishiura, M. Tanaka, J. Rohacova, Y. Nakagawa, Y. Funada, K. Koike, Y. Yamamoto, S. Shishido, T. Kojima, T. Saeki, T. Ozeki and O. Ishitani, *J. Am. Chem. Soc.*, 2013, **135**, 13266-13269.

2. (a) S. Tanaka, Y. Matsubara, T. Asatani, T. Morimoto, O. Ishitani and K. Onda, *Chem. Phys. Lett.*, 2016, **662**, 120-126; (b) Y. Shimoda, K. Miyata, M. Funaki, T. Ehara, T. Morimoto, S. Nozawa, S.-i. Adachi, O. Ishitani and K. Onda, *Inorg. Chem.*, 2021, **60**, 7773-7784.

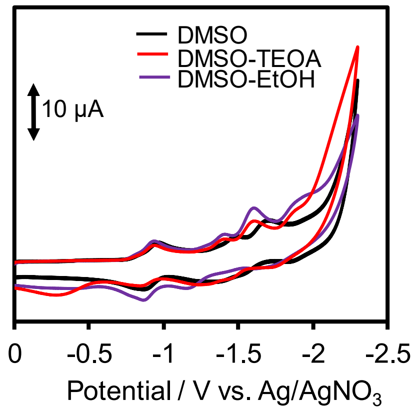

Figure S5. Cyclic voltammograms of $(TBA^{+})_4$(**GePOM^{4-}**) measured under CO_2 atmosphere: in DMSO, DMSO-EtOH (5:1 v/v), and DMSO-TEOA (5:1 v/v) solutions. $(TBA^{+})(PF_6)$ was added as supporting electrolyte: a glassy carbon working electrode, 0.01 mM $Ag/AgNO_3$ reference electrode, and a Pt counter electrode with scan rate of 100 mV s^{-1}.

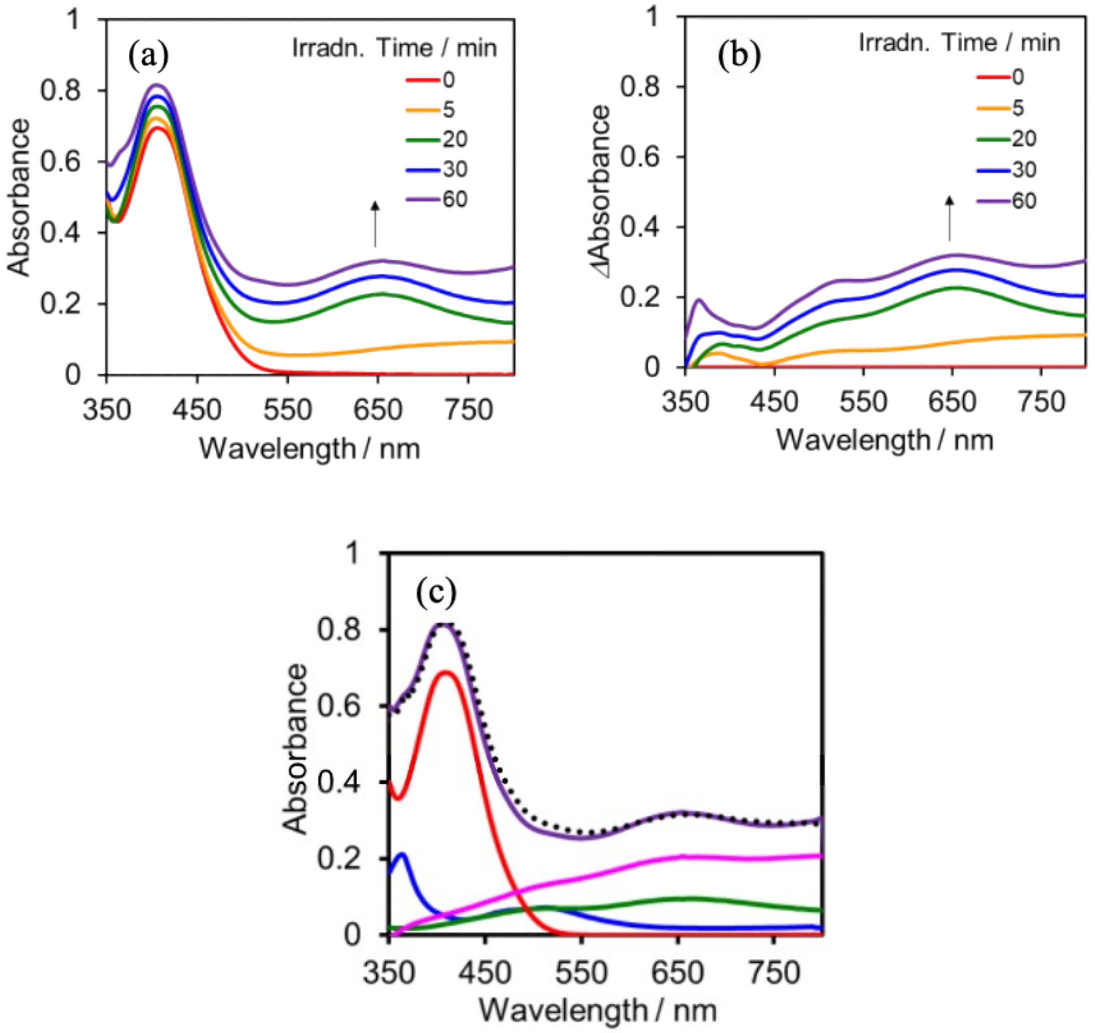

Figure S6. (a) UV-vis absorption spectra of the DMSO-TEOA (5:1 v/v) solution containing (**Ring^{4+})-(GePOM^{4-}**) (0.05 mM) during irradiation. The DMSO-TEOA (5:1 v/v) solution containing (**Ring^{4+})-(GePOM^{4-}**) (0.05 mM) was irradiated at λ_{ex} = 436 nm (light intensity: 5 x 10^{-9} einstein s^{-1}) under a CO_2 atmosphere, and (b) spectral changes and their deference spectra between before and after irradiation: irradiation time 5 min (orange line), 20 min (green line), 30 min (blue line), 60 min (purple line). (c) UV-vis absorption spectrum after irradiation for 60 min and its fitting result using the spectra of **Ring^{4+}** (red line), **GePOM^{6-}** (green line), **H_2GePOM^{6-}** (pink line), and **Ring^{3+}** (blue line).

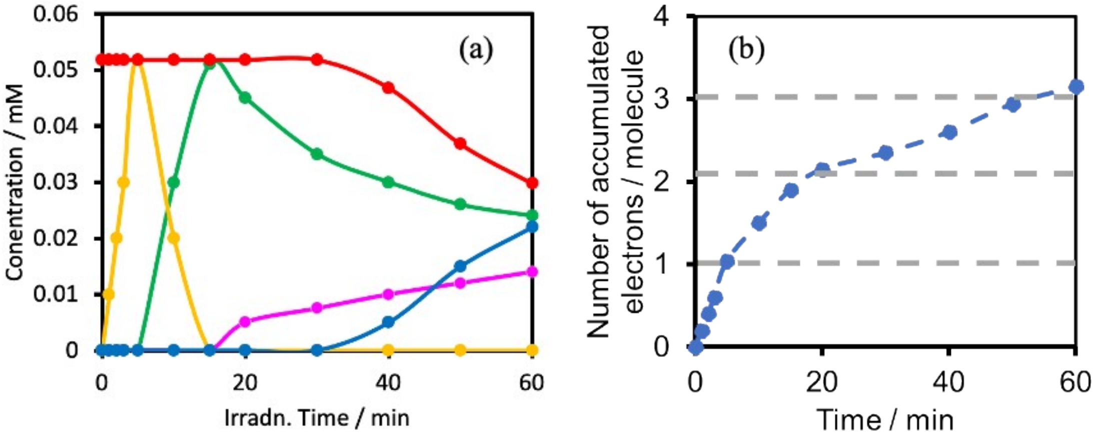

Figure S7. (a) Concentrations of the **Ring^{4+}** (red line) and **GePOM^{4-}** (black line) units and the reduced species of each unit: **GePOM^{5-}** (orange line), **GePOM^{6-}** (green line), **H_2GePOM^{6-}** (pink line), and **Ring^{3+}** (blue line). (b) Accumulated electrons in one molecule of (**Ring^{4+})-(GePOM^{4-}**).

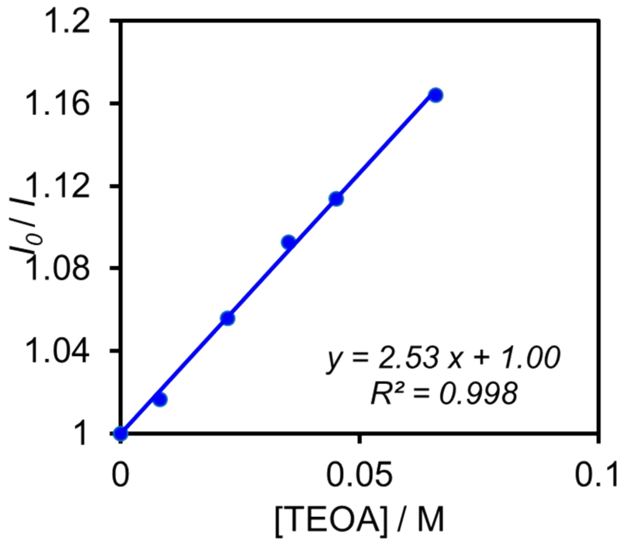

Figure S8. Stern-Volmer plots of (**Ring^{4+})-(SiPOM^{4-}**) obtained from emission strength changes (I_0/I) by addition of TEOA into a DMSO solution at 25 ˚C under Ar. The excitation wavelength was 400 nm. For reference, the Stern-Volmer constant of **Ring^{4+}** by TEOA was reported as K_{SV} = 1.26 M^{-1}.[4]

Investigation of spectral changes of the reaction solution during irradiation with high light intensity

Figure S8a shows the UV-vis absorption spectra of the photocatalytic reaction solution during irradiation at λ_{ex} = 436 nm (2.5 x 10^{-7} einstein s^{-1}) in the systems using **Ring^{4+}** as the PS. Immediately after starting irradiation, new absorption bands appeared in a wide range of visible regions with absorption maxima at ~550 nm and ~860 nm, and they slowly decreased after 30 min of irradiation. These spectral changes can be attributed to the formation of Ru polymer, as shown in Eq. S3. Further irradiation caused a decrease in both Ru polymers and **Ring^{4+}**, which was synchronised with the decline in photocatalysis (Figure 12c).

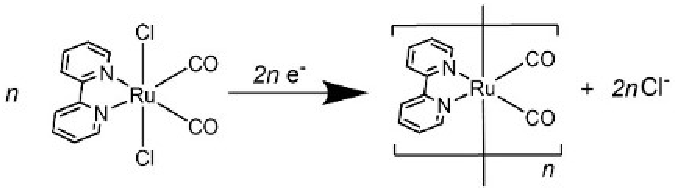

(S3)

In the initial stage of the photocatalytic reaction using (**Ring^{4+})-(SiPOM^{4-}**) as the PS (Figure S8b), Ru polymer was also produced, but its formation yield was lower than that using **Ring^{4+}**. In addition, the Ru polymer disappeared upon irradiation for 3 h, and the spectrum indicates that TWERS [(**Ring^{4+}**)-(**SiPOM^{6-}**)]$^{2-}$ accumulated. Notably, the photocatalytic activity of this system did not decrease even after 3 h of irradiation (Figure 12a).

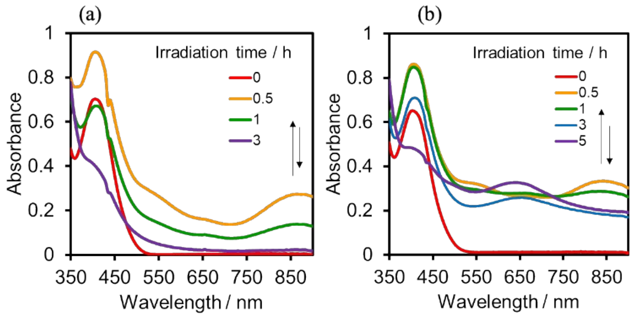

Figure S8. UV-vis absorption spectra of the photocatalytic reactions using (a) **Ring^{4+}** or (b) (**Ring^{4+})-(SiPOM^{4-}**) as PS. The reaction conditions are described in Figure 12.

It was reported that, in the photocatalytic reduction of CO_2 using **RuCAT**, the Ru dimer $(Ru(bpy)(CO)_2Cl)_2$ is formed via the detachment of one of the Cl^- ligands from the OERS of **RuCAT**, and further reduction of the Ru dimer induces the formation of a black polymer.[1-3] These results and investigations suggest that TWERS [(**Ring^{4+}**)-(**SiPOM^{6-}**)]$^{2-}$ suppresses the formation of the Ru polymer during the photocatalytic reaction and increases the durability of the photocatalyst. TWERS should efficiently give an electron to intermediate(s) made from the OERS of **RuCAT** for CO_2 reduction to suppress the

polymerisation of the Ru catalyst.

References

1. J.-M. Lehn and R. Ziessel, *J. Orgmet. Chem.*, 1990, **382**, 157-173.
2. Y. Kuramochi, J. Itabashi, K. Fukaya, A. Enomoto, M. Yoshida and H. Ishida, *Chem. Sci.* 2015, **6**, 3063-3074.
3. Y. Kuramochi, O. Ishitani and H. Ishida, *Coord. Chem. Rev.*, 2018, **373**, 333-356.

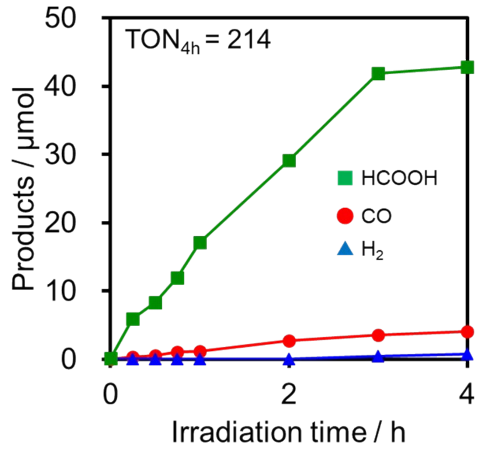

Figure S9. Formation of products during irradiation to DMSO-TEOA (5:1 v/v) solutions containing **Ring^{4+}** (0.05 mM) and **RuCAT** (0.05 mM) at l_{ex} = 436 nm (5.0 x 10^{-9} einstein s^{-1}) under CO_2 atmosphere.

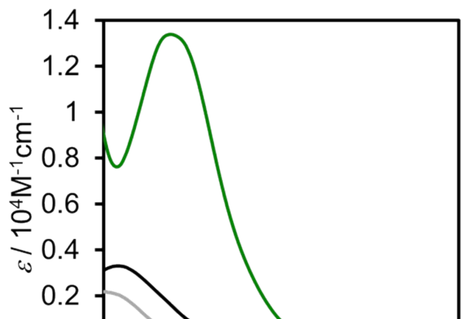

Figure S10. UV-vis absorption spectra of (**Ring^{4+})-(SiPOM^{4-}**) (green), **ReCAT** (black), and **RuCAT** (gray) in DMSO-TEOA (5:1 v/v) solutions.

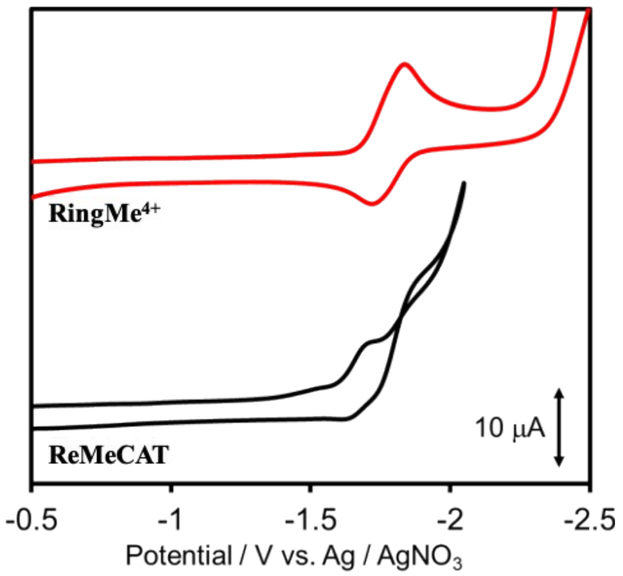

Figure S11. Cyclic Voltammograms of DMSO-TEOA (5:1 v/v) solutions containing complexes, i.e., (**RingMe^{4+}**)(PF_6)$_4$ and *fac*-[Re(Me_2bpy)(CO_3){OC(O)OC_2H_4N(C_2H_4OH)$_2$}] (**ReMeCAT**), and (TBA^+)(PF_6) as supporting electrolyte measured under CO_2 atmosphere by using a glassy carbon working electrode, 0.01 mM Ag/$AgNO_3$ reference electrode, and a Pt counter electrode with scan rate of 100 mV s^{-1}.

Photocatalytic CO_2 reduction using (RingMe^{4+})-(SiPOM^{4-}) as PS and *fac*-[Re(Me_2bpy)(CO_3){OC(O)OC_2H_4N(C_2H_4OH)$_2$}] (ReMeCAT, Me_2bpy = 4,4'-dimethyl-2,2'-bipyridine) as a catalyst.

The synthesis of (**RingMe^{4+})-(SiPOM^{4-}**) can be achieved using the same method as (**RingMe^{4+})-(SiPOM^{4-}**), except for (**RingMe^{4+}**)(PF_6)$_4$ instead of (**Ring^{4+}**)(PF_6)$_4$. However, the ^{1}MLCT absorption band and emission of (**RingMe^{4+})-(SiPOM^{4-}**) were slightly blue-shifted (λ_{ab} = 390 nm, λ_{em} = 580 nm), the oxidative quenching of emission of the **RingMe^{4+}** unit by the **SiPOM^{4-}** unit was also observed in approximately 90%

yield. The UV-vis absorption spectral changes

A DMSO-TEOA (5:1 v/v) solution containing **(RingMe^{4+})-(SiPOM^{4-})** and **ReMeCAT** (0.05 mM each) was irradiated at λ_{ex} = 436 nm with a low light intensity (5.0 x 10^{-9} einstein s^{-1}) under a CO_2 atmosphere, also giving CO selectively (Table S3). The TON_{CO} for irradiation for 12 h was 43. Because TON_{CO} was 2.2 in the absence of **(RingMe^{4+})-(SiPOM^{4-})**, the mixed systems of **(RingMe^{4+})-(SiPOM^{4-})** as the PS and **ReMeCAT** as the catalyst should work well as a photocatalytic system for CO_2 reduction. Figure S12 shows the UV-vis absorption changes of the photocatalytic reaction solution and the number of accumulated electron(s) in one molecule of **(RingMe^{4+})-(SiPOM^{4-})** after 60-min of irradiation. These results clearly indicate that two electrons were accumulated in **(RingMe^{4+})-(SiPOM^{4-})** in the photostational state of the photocatalytic reaction, that is, the TWERS [**(RingMe^{4+})-(SiPOM^{6-})**]$^{2-}$ cannot reduce the intermediate produced from the OERS of **ReMeCAT**. Therefore, we can conclude that the intermediate has a more negative potential than $E_{1/2}^{red}$ = -1.40 V.

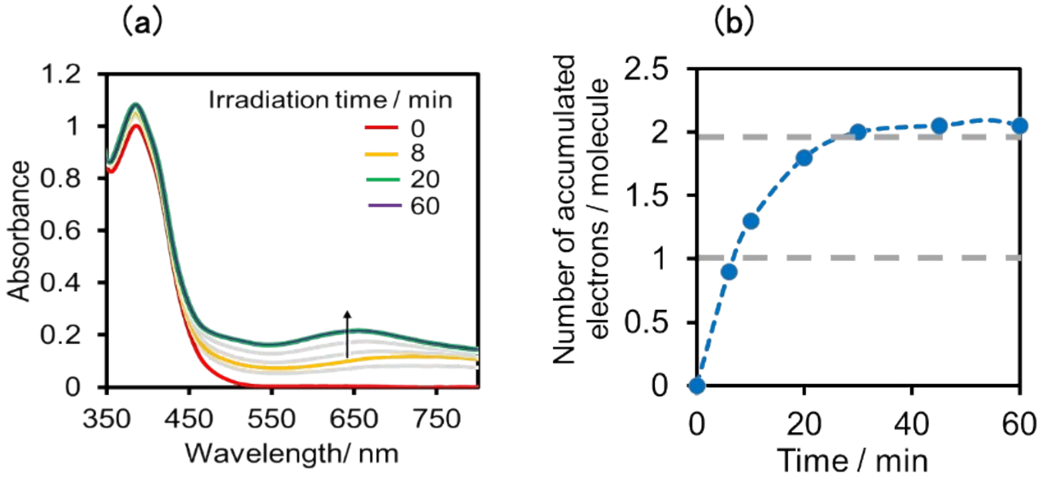

Figure S12. (a) UV-vis absorption spectra of the DMSO-TEOA (5:1 v/v) solution containing **(RingMe^{4+})-(SiPOM^{4-})** (0.05 mM) and **ReMeCAT** (0.05 mM) during irradiation at λ_{ex} = 436 nm under a CO_2 atmosphere, and (b) accumulated electrons in one molecule of **(RingMe^{4+})-(SiPOM^{4-})**.

Table S4. Photocatalytic reactions using **ReMeCAT**[a]

Photosensitizer	Catalyst	Time	TON_{CO}	
		h	CO	HCOOH
**(RingMe^{4+})-(SiPOM^{4-})**	**ReMeCAT**	12	43	trace
**(Ring^{4+})-(SiPOM^{4-})**	**ReMeCAT**	12	2	trace

[a] DMSO-TEOA (5:1 v/v) solutions containing PS (0.05 mM) and/or **RuMeCAT** (0.05

mM) were irradiated at λ_{ex} = 436 nm (5.0 x 10^{-9} einstein s^{-1}) under CO_2 atmosphere.

EXPERIMENTAL SECTION

1. Material Characterization

The Nuclear magnetic resonance spectroscopy (^{1}H-NMR) spectra were recorded on a JEOL ECX400-II spectrometer (400 MHz), DMSO-d_6 was used as a solvent, and its residual peaks were used as an internal standard. The Fourier-transform infrared spectroscopy (FT-IR) spectra were measured on a JASCO FT/IR-610 spectrometer with a TGS detector (resolution: 1 cm^{-1}) and GL Science SL-CaF_2 cell (pass length: 0.5 mm). Emission spectra (ES) were recorded using a JASCO FP-6500 spectrometer. Emission lifetimes were measured with a Horiba NAES-1100 time-correlated single-photon-counting system (the excitation source was an NFL-111nanosecond H_2 lamp, and the instrument response was less than 1 ns). The emission quantum yields were measured using a Quantaurus-QY Plus C13534-01 quantum yield analyzer (HAMAMATSU) with a 150 W xenon arc lamp light source (400 nm) and an A10080-01 monochromator.

2. Electrochemical analysis

Cyclic voltammograms (CVs) of the solutions containing the substrate (0.5 mM) and $(TBA^+)(PF_6)$ (0.1 M) as the electrolyte were recorded using an ALS/CHI BAS CHI760Es electrochemical analyzer with a glassy carbon working electrode (ϕ =3 mm), silver/silver nitrate ($Ag/AgNO_3$, 0.01 M) reference electrode, and platinum (Pt) wire counter electrode. The supporting electrolyte was dried in vacuo at 100 °C for 1 d before use. Details of flow electrolysis experiments have been described elsewhere: we used following equipment: ALS/CHI BAS CHI-720D electrochemical analyzer, a JASCO PU-980 flow pump, a VF-2 electrolysis cell (EC Frontier) with carbon felt working electrode for electrolysis, a quartz flow cell (1.5 mm light path) for spectrum measurements, a $Ag/AgNO_3$ (0.01 M) reference electrode, a Pt wire counter electrode, and a Photal MCPD-9800 photodiode array spectrometer with a D_2/I_2 mixed lamp. A solution containing the substrates (0.5 mM) and $(TBA^+)(PF_6)$(0.1 M) was bubbled with CO_2 for 20 min and then transferred at a flow rate of 0.11 ml min^{-1}. [1] Dynamic light scattering was measured using a Horiba SZ-100 nanoparticle analyzer with λ_{det} = 642 nm, scattering angle = 90° or 173°, T = 25 °C. The particle sizes of the substrates were calculated using a viscosity coefficient of DMSO = 1.98 mPa s.

3. Materials

DMSO (special grade, Wako Chemicals) was dried on CaH_2 under an Ar atmosphere for one night, and then distilled under reduced pressure at ~ 80 °C, which was used within one week. TEOA (special grade, Kanto Chemicals) was distilled under an Ar atmosphere

at reduced pressure at 130~ 150 °C. The distilled TEOA was kept under an Ar atmosphere and be used within one month. $(TBA^+)(PF_6)$ was recrystallized with hexane–ethyl acetate mixed solutions twice, and then dried at 100 °C under reduced pressure for one night just before use. The other supplied chemicals were used without any purification.

$(TBA^+)_4$(**SiPOM**$^{4-}$),[2] $(TBA^+)_4$(**GePOM**$^{4-}$),[3] (**Ring**$^{4+}$)$(PF_6)_4$,[4] *fac*-[Re(bpy)$(CO)_3$(MeCN)](PF_6),[5] *fac*-[Re(Me_2bpy)$(CO)_3$(MeCN)](PF_6) [6], and **RuCAT**[7] were synthesized according to the reported procedures. **Re-CAT** and **ReMeCAT** were synthesized from *fac*-[Re(bpy)$(CO)_3$(MeCN)](PF_6), and *fac*-[Re(Me_2bpy)$(CO)_3$(MeCN)](PF_6), respectively, according to the reported procedures. [6]

4. Synthesis

[Re(Me_2bpy)$(CO)_3$(μ_2-dpph)Re(Me_2bpy)$(CO)_3$]$(PF_6)_2$

A tetrahydrofuran solution (60 mL) containing *fac*-[Re(Me_2bpy)$(CO)_3$(OTf)] (401.0 mg, 0.66 mmol) and dpph (150.7 mg; 0.33 mmol) was refluxed under Ar atmosphere in the dark for 2 days. The precipitate was filtered and washed with tetrahydrofuran (THF) and diethyl ether. The solid was dissolved in a small amount of methanol and another methanol solution saturated with ammonium hexafluorophosphate (NH_4PF_6) was added. The precipitated yellow solid was then filtered and washed with diethyl ether. Yield: 94% (513.94 mg). FT-IR (CH_2Cl_2, cm^{-1}): 2036(ν_{CO}), 1949(ν_{CO}), and 1920(ν_{CO}). ESI-MS (CH_3CN, *m/z*): 682 $[M–2PF_6]^{2+}$.

[Re(Me_2bpy)$(CO)_3${(μ_2-dpph)Re$(CO)_2$(Me_2bpy)}$_2$(μ_2-dpph)Re(Me_2bpy)$(CO)_3$]$(PF_6)_4$

A dichloromethane solution (300 mL) containing 300 mg (0.19 mmol) of [Re(Me_2bpy)$(CO)_3$(μ_2-dpph)Re(Me_2bpy)$(CO)_3$]$(PF_6)_2$ was irradiated under N_2 atmosphere using a high-pressure Hg lamp (Eikosha Co. Ltd) for 30 min. After evaporation of the solvent, diethyl ether was added to obtain a precipitate. The precipitate was dissolved into 40 mL of dichloromethane with dpph (37.0 mg; 0.093 mmol), and the solution was refluxed for 25 h under dim conditions. After evaporation of the solvent, the residue was separated using size-exclusion chromatography (JAI LC-9201 preparative recycle HPLC apparatus with two sequentially connected Shodex PROTEIN KW-2002.5 columns (300 mm × 20.0 mm i.d.), a KW-LG guard column (50 mm × 8.0 mm i.d.), and a JASCO 870-UV detector, eluent: a 1:1 (v/v) mixture of MeOH and acetonitrile (MeCN) containing 0.15 M ammonium acetate ($CH_3CO_2NH_4$). The fractions containing the target complex were evaporated, and the residue was extracted twice using dichloromethane and water to remove $CH_3CO_2NH_4$. The solid obtained by evaporation of the

dichloromethane phase was dissolved in a small amount of methanol, and a saturated methanol solution with NH_4PF_6 and water was added. The precipitate was filtered and dried at 60 °C under a vacuum. Yield: 53.9 % (179.86 mg). **^{1}H-NMR** (300 MHz, acetone-d_6) / ppm: 8.77 (d, 4H, J = 5.8 Hz, tri-Me$_2$bpy-6), 8.39 (s, 4H, tri-Me$_2$bpy-3), 8.17 (s, 4H, bis-Me$_2$bpy-3), 8.12 (d, 4H, J = 5.8 Hz, tri-bpy-6), 7.53-7.15 (m, 76H, bis-Me$_2$bpy-3, tri-Me$_2$bpy-5, Ph-*p*, Ph-*m*, Ph-*o*), 6.79 (d, 4H, J = 6.0 Hz, bis-Me$_2$bpy-5), 2.59 (s, 12H, tri-C$\underline{H}_3$), 2.46 (s, 12H, bis-C$\underline{H}_3$), 1.92-1.82 (m, 12H, PPh$_2$-C$\underline{H}_2$- (CH$_2$)$_4$-C$\underline{H}_2$-PPh$_2$), 1.15-0.90 (m, 24H, PPh$_2$-CH$_2$-(C$\underline{H}_2$)$_4$-CH$_2$-PPh$_2$). **FT-IR** (CH_3CN, cm^{-1}): 2037(ν_{CO}), 1948(ν_{CO}), 1930(ν_{CO}), 1921(ν_{CO}), 1851(ν_{CO}). **ESI-MS** (CH_3CN, *m/z*): 781 [M - 4PF$_6$]$^{4+}$, 1090 [M - 3PF$_6$]$^{3+}$.

[{Re(bpy)(CO)$_2$(μ_2-dpph)}$_4$][SiW$_{12}$O$_{40}$] (**(Ring^{4+})-(SiPOM^{4-})**)

(**Ring^{4+}**)(PF$_6$)$_4$ (12.0 mg, 3.01 μmol) was dissolved in an acetonitrile solution (8 ml), and another acetonitrile solution (6 ml) containing (TBA$^+$)$_4$(**SiPOM^{4-}**) 11.5 mg (3.0 μmol) was added with strong stirring at room temperature. The produced yellow solids were filtered and washed with acetonitrile: yield 96% (18.2 mg). ^{1}H-NMR (400MHz, DMSO-d_6) / ppm: 8.30 (d, 8H, J = 9.2 Hz, bpy-6), 8.17 (br, 8H, bpy-3), 7.97 (t, 8H, J = 8.4 Hz, bpy-5), 7.21 (t, 12H, J = 7.1 Hz, Ph-p), 7.13 (t, 24H, J = 6.8 Hz, Ph-m), 6.99 (br, 32H, Ph-o, bpy-4), 1.81 – 1.69 (br, 16H, PPh$_2$-C$\underline{H}_2$-CH$_2$-CH$_2$-CH$_2$-CH$_2$-C$\underline{H}_2$-PPh$_2$), 0.96 – 0.71 (m, 32H, PPh$_2$-CH$_2$-C$\underline{H}_2$-C$\underline{H}_2$-C$\underline{H}_2$-C$\underline{H}_2$-CH$_2$-PPh$_2$). FT-IR (KBr, cm^{-1}): 1928 (ν_{CO}), 1853 (ν_{CO}), 970 ($\nu_{W=O}$), 922 ($\nu_{W\text{-}O\text{-}W}$), 884 ($\nu_{Si\text{-}O}$), 805 ($\nu_{W\text{-}O\text{-}W}$). Anal. Calcd for $C_{168}H_{160}N_8O_{48}P_8Re_4SiW_{12}$: C, 32.10; H, 2.57; N, 1.78. Found C, 31.87; 101 H, 2.72; N, 1.68.

[{Re(bpy)(CO)$_2$(μ_2-dpph)}$_4$][GeW$_{12}$O$_{40}$] •6H$_2$O (**(Ring^{4+})-(GePOM^{4-})**)

The same procedures for **(Ring^{4+})-(SiPOM^{4-})** were applied except for the use of (TBA$^+$)$_4$(**GePOM^{4-}**) instead of (TBA$^+$)$_4$(**SiPOM^{4-}**). (TBA$^+$)$_4$(**GePOM^{4-}**) (15.5 mg, 4.0 μmol) and (**Ring^{4+}**)(PF$_6$)$_4$ 15.0 mg (3.8 μmol) were used. Yield: 97% (23.2 mg). ^{1}H-NMR (400MHz, DMSO-d_6) / ppm: 8.30 (d, 8H, J = 8.2 Hz, bpy-6), 8.20-8.12 (br, 8H, bpy-3), 7.96 (t, 8H, J = 7.3 Hz, bpy-5), 7.22 (t, 12H, J = 7.2 Hz, Ph-p), 7.14 (t, 24H, J = 7.2 Hz, Ph-m), 7.06-6.91 (br 32H, J = 5.5, 7.3 Hz, Ph-o, bpy-4), 1.89 – 1.63 (br, 16H, PPh$_2$-C$\underline{H}_2$-CH$_2$-CH$_2$-CH$_2$-CH$_2$-C$\underline{H}_2$-PPh$_2$), 0.94 – 0.68 (m, 32H, PPh$_2$-CH$_2$-C$\underline{H}_2$-C$\underline{H}_2$-C$\underline{H}_2$-C$\underline{H}_2$-CH$_2$-PPh$_2$). FT-IR (KBr, cm^{-1}): 1925(ν_{CO}), 1852(ν_{CO}), 969($\nu_{W=O}$), 921($\nu_{W\text{-}O\text{-}W}$), 882($\nu_{Ge\text{-}O}$), 804($\nu_{W\text{-}O\text{-}W}$). Anal. Calcd for $C_{168}H_{172}GeN_8O_{54}P_8Re_4W_{12}$: Calcd for C, 31.34; H, 2.69; N, 1.74. Found: C, 31.36; H, 2.77; N, 1.68.

$[\{Re(Me_2bpy)(CO)_2(\mu_2\text{-dpph})\}_4][SiW_{12}O_{40}]$ (**(Ring(tmb)$^{4+}$)-(SiPOM^{4-})**)

The same procedures for **(Ring^{4+})-(SiPOM^{4-})** were applied except for the use of (**RingMe^{4+}**)$(PF_6)_4$ instead of (**Ring^{4+}**)$(PF_6)_4$. $(TBA^+)_4$(**SiPOM^{4-}**) (19.6 mg, 5.1 μmol) and (**Ring(tmb)$^{4+}$**)$(PF_6)_4$ (20.5 mg, 5.0 μmol) were used. Yield: 97% (31.0 mg). FT-IR (KBr, cm^{-1}): 1926(ν_{CO}), 1852(ν_{CO}), 968($\nu_{W=O}$), 919($\nu_{W\text{-}O\text{-}W}$), 882($\nu_{Si\text{-}O}$), 805($\nu_{W\text{-}O\text{-}W}$). Anal. Calcd for $C_{176}H_{176}N_8O_{48}P_8Re_4SiW_{12}$: C, 33.04; H, 2.77; N, 1.75. Found: C, 32.78; H, 2.88; N, 1.63. Anal. Calcd for $C_{168}H_{172}GeN_8O_{54}P_8Re_4W_{12}$: C, 31.34; H, 2.69; N, 1.74. Found: C, 31.36; H, 2.77; N, 1.68.

^{1}H NMR of (**Ring^{4+}**)$(PF_6)_4$

^{1}H-NMR (400MHz, DMSO-d_6) / ppm: 8.33（d, 8H, J = 8.4 Hz, bpy-6）, 8.11（d, 8H, J = 4.4 Hz, bpy-3）, 7.88（t, 8H, J = 7.8 Hz, bpy-5）, 7.30（t, 12H, J = 7.2 Hz, Ph-p）, 7.20（t, 24H, J = 7.2 Hz, Ph-m）, 7.05（dd, 24H, J = 6.0, 7.2 Hz, Ph-o）, 6.59（t, 8H, J = 6.4 Hz, bpy-4）, 1.91 - 1.74 (br, 16H, $PPh_2\text{-}C\underline{H}_2\text{-}CH_2\text{-}CH_2\text{-}CH_2\text{-}CH_2\text{-}C\underline{H}_2\text{-}PPh_2$), 1.01 - 0.74 (br, 32H, $PPh_2\text{-}CH_2\text{-}C\underline{H}_2\text{-}C\underline{H}_2\text{-}C\underline{H}_2\text{-}C\underline{H}_2\text{-}CH_2\text{-}PPh_2$).

5. X-ray crystallography

Preparation of Single Crystals of **(Ring^{4+})-(SiPOM^{4-})** and (**Ring^{4+}**)$(PF_6)_4$

(Ring^{4+})-(SiPOM^{4-}) was recrystallized using *N,N*-dimethylacetamide/methanol to obtain single crystals suitable for single-crystal structural analysis. The (**Ring^{4+}**)$(PF_6)_4$ was recrystallized using ethanol/hexane to obtain single crystals suitable for single-crystal structure analysis.

Crystal structure determination

Crystallographic data for **(Ring^{4+})-(SiPOM^{4-})** were collected at 123 K using a Rigaku XtaLAB PRO diffractometer equipped with a PILATUS 200 K hybrid pixel array detector with Mo Kα (λ = 0.71075 Å). The diffraction profiles were integrated using a CrysAlisPro v171.41.93a (Rigaku OD, 2020). The crystal data were solved using direct methods using the SHELXT program and refined with SHELXL, [8, 9] and highly disordered solvent molecules were treated by PLATON/SQUEEZE. [10] Anisotropic thermal parameters were used to refine all non-H atoms.

Crystallographic data for (**Ring^{4+}**)$(PF_6)_4$ were collected at 123 K on a Rigaku XtaLAB PRO diffractometer equipped with a PILATUS 200 K hybrid pixel array detector with Mo Kα (λ = 0.71075 Å). Diffraction profiles were integrated using CrysAlisPro

v171.39.20a (Rigaku OD, 2015). The crystal data were solved by directed methods using the SHELXT program and were refined with SHELXL.[8, 9] Highly disordered PF_6 anion and solvent molecules were treated by PLATON/SQUEEZE.[10] Anisotropic thermal parameters were used to refine all non-H atoms.

The crystallographic data were summarized in Table 1, and CCDC 2153829 and 2153830 contain the Supporting crystallographic data, data of which can be obtained free of charge from The Cambridge Crystallographic Data Centre via www.ccdc.cam.ac.uk/structures/.

6. Photocatalytic reactions and measurements of UV-vis absorption spectra during irradiation

A DMSO-TEOA (5:1 v/v) solution (4 mL) containing the complex was introduced into a quartz cell (1 × 1 × 4 cm) and bubbled with CO_2 for 20 min. The sealed cell was irradiated at 436 nm with stirring using a 300-W high-pressure Hg lamp (Ushio Co., USH-500SC) equipped with a 436-nm bandpass filter (Asahi Spectra Co.), ND filters (Chuo Precision Industrial Co.), and a 5-cm long $CuSO_4$ solution (20 g L^{-1}) filter. The reaction temperatures of the solutions were maintained at 25 °C using an IWAKI constant temperature system CTS-134A. A $K_3Fe(C_2O_4)_3$ actinometer determined the incident light intensity. UV-vis absorption spectra during the photochemical reactions were measured *in situ* on an MCPD-6800 photodiode array spectrometer and a D_2-I_2 mixed lamp (Otsuka Electronic Co.). The irradiated samples were kept in the dark for 3 h to achieve gas-liquid equilibrium, and then the gas phase was analyzed by gas chromatography with a thermal conductivity detector. HCOOH formation was analyzed using a CAPI-3300I capillary electrophoresis system (Otsuka Electronics Co.). The quantum yield (Φ) and turnover number (TON) of the products were defined as [formed product / mol]/[absorbed photon / einstein] and [formed CO / mol]/[PS / mol], respectively, when the concentration of the catalyst was same as that of PS.

References

1. K. Ozawa, Y. Tamaki, K. Kamogawa, K. Koike and O. Ishitani, *J. Chem. Phy.*, 2020, **153**, 154302.

2. A. Teze and G. Herve, *Inorg. Synth.*, 1990, **27**, 85-96.

3. C. Rocchiccioli-Deltcheff, M. Fournier, R. Franck and R. Thouvenot, *Inorg. Chem.*, 1983, **22**, 207-216.
4. T. Asatani, Y. Nakagawa, Y. Funada, S. Sawa, H. Takeda, T. Morimoto, K. Koike and O. Ishitani, *Inorg. Chem.*, 2014, **53**, 7170-7180.
5. J. V. Casper and T. J. Meyer, *J. Phys. Chem.*, 1983, **87**, 952-957.
6. H. Kumagai, T. Nishikawa, H. Koizumi, T. Yatsu, G. Sahara, Y. Yamazaki, Y. Tamaki and O. Ishitani, *Chem Sci*, 2019, **10**, 1597-1606.
7. M. Haukka, J. Kiviaho, M. Ahlgren and T. A. Pakkanen, *Organometallics*, 1995, **14**, 825-833.
8. G. M. Sheldrick, *Acta Crystallogr. A*, 2008, **64**, 112-122.
9. G. M. Sheldrick, *Acta C Crystallogr. C*, 2015, **71**, 3-8.
10. A. L. Spek, *Acta Crystallogr. C*, 2015, **71**, 9-18.
